# Solubilization and enhanced degradation of benzene phenolic derivatives—Bisphenol A/Triclosan using a biosurfactant producing white rot fungus *Hypocrea lixii* S5 with plant growth promoting traits

**DOI:** 10.3389/fmicb.2024.1433745

**Published:** 2024-09-18

**Authors:** Mridula Chaturvedi, Navpreet Kaur, Pattanathu K. S. M. Rahman, Shashi Sharma

**Affiliations:** ^1^Amity Institute of Biotechnology, Amity University, Noida, UP, India; ^2^Centre for Natural Products and Discovery, School of Pharmacy and Biomolecular Sciences, Liverpool John Moores University, Liverpool, United Kingdom

**Keywords:** lipopeptide, lignin-modifying enzymes, degradation correlation, metabolic pathway, bioaccumulation

## Abstract

**Introduction:**

Endocrine disrupting chemicals (EDCs) as benzene phenolic derivatives being hydrophobic partition to organic matter in sludge/soil sediments and show slow degradation rate owing to poor bioavailability to microbes.

**Methods:**

In the present study, the potential of a versatile white rot fungal isolate S5 identified as *Hypocrea lixii* was monitored to degrade bisphenol A (BPA)/triclosan (TCS) under shake flask conditions with concomitant production of lipopeptide biosurfactant (BS) and plant growth promotion.

**Results:**

Sufficient growth of WRF for 5 days before supplementation of 50 ppm EDC (BPA/TCS) in set B showed an increase in degradation rates by 23% and 29% with corresponding increase in secretion of lignin-modifying enzymes compared to set A wherein almost 84% and 97% inhibition in fungal growth was observed when BPA/TCS were added at time of fungal inoculation. Further in set B, EDC concentration stimulated expression of laccase and lignin peroxidase (Lip) with 24.44 U/L of laccase and 281.69 U/L of Lip in 100 ppm BPA and 344 U/L Lip in 50 ppm TCS supplemented medium compared to their respective controls (without EDC). Biodegradation was also found to be correlated with lowering of surface tension from 57.02 mN/m (uninoculated control) to 44.16 mN/m in case of BPA and 38.49 mN/m in TCS, indicative of biosurfactant (BS) production. FTIR, GC-MS, and LC-ESI/MSMS confirmed the presence of surfactin lipopeptide isoforms. The WRF also displayed positive plant growth promoting traits as production of ammonia, indole acetic acid, siderophores, Zn solubilization, and 1-1-aminocyclopropane-1-carboxylate (ACC) deaminase activity, reflecting its soil restoration ability.

**Discussion:**

The combined traits of biosurfactant production, EDC degradation and plant growth promotion displayed by WRF will help in emulsifying the hydrophobic pollutants favoring their fast degradation along with restoration of contaminated soil in natural conditions.

## 1 Introduction

The increased industrialization and urbanization have resulted in the release of hazardous compounds which contaminate air, water, and soil. These have been collectively categorized under endocrine disrupting chemicals (EDCs) and have been reported to interfere with the normal functioning of the endocrine system in humans and animals (Marlatt et al., 2022). These comprise of the recalcitrant man-made compounds used in plasticizers, flame retardants, surfactants, pesticides, pharmaceuticals, personal care products, etc. Out of the total 85,000 chemicals known till date, only more than thousands have been classified as EDCs, while the others remain unidentified due to unsatisfactory analyses leading to insufficient information about their toxic effects (Chaturvedi et al., 2023).

Among the most common EDCs occurring in soil/waste waters are the phenolic derivatives of benzene—bisphenol A (BPA) and triclosan (TCS) as these are the basic raw materials extensively used for daily use consumer items. Bisphenol A or 4,4′-dihydroxy-2,2-diphenylpropane (C_15_H_16_O_2_) is used for the production of polycarbonate plastics, flame retardants, thermal paper color developer, epoxy resins, lubricants, paints, etc. (Chaturvedi et al., 2024). Similarly, triclosan or 5-chloro-2-(2,4-dichlorophenoxy) phenol (C_12_H_7_Cl_3_O_2_) is used as a broad-spectrum antimicrobial agent in pharmaceutical and personal care products at a concentration range of 0.1%−0.45% (w/v) (Wang et al., 2024). Because of the applications, their market size is always high with 7.96 million metric tons production of BPA and 1,500 tons of TCS in 2024 (Manzoor et al., 2022; Milanović et al., 2023). The high production rate of these raw materials has resulted in the average concentration of BPA with 29.19 ng/g, 30.21ng/L, and 610.09 ng/gdw in soil, irrigation water, and sediments, respectively, having half-life in water as 38 days, 75 days (soil), and 340 days (sediment) (Qin et al., 2024). Similarly, the worldwide concentration of TCS was reported as 0.001–40 μg/L in surface waters, 0.02–86.161 μg/L in wastewater influents, 0.023–5.370 μg/L in effluents from waste treatment plants, and 0.201–328.8 μg/L in ground water sources (Dar et al., 2022). The half-life of TCS ranges from 1.3 to 1.4 days (water) and 53.7 to 60.3 days (sediments). The high tendency for sorption to soil and sediments of these EDCs is due to the high octanol-water partitioning coefficient (log KOW) being 3.32 for BPA and 4.76 for TCS which decreases the bioavailability to microbes for degradation (Milanović et al., 2023). In soil, these alter the soil moisture, soil temperature, and soil micro-organisms and degrade the physical soil structure in terms of pore size, bulk density, and air permeability (Maddela et al., 2022). In plants, these cause oxidative damage by decreasing antioxidant enzyme activity (Chaturvedi et al., 2023). Thus, these EDCs make their way to the highest trophic level favoring bioaccumulation at each trophic level via food chain. In humans and animals, EDCs gain their entry directly or indirectly via inhalation, ingestion, or dermal exposure and cause multiple health disorders (Milanović et al., 2023).

The negative effects of these benzene phenolic derivatives on the ecosystem necessitate the adoption of removal methods with formation of non-toxic metabolites. Compared to physical, chemical, and physiochemical methods having high capital/energy costs, plant modification, and increased sludge, microbe-mediated bioremediation offers advantages as microbes secrete catalytic enzymes that assist in eco-friendly degradation of complex structures (Nishat et al., 2023). Numerous studies using white rot fungus as *Phanerochaete, Pleurotus, Trametes*, etc. have been reported for degradation of xenobiotic pollutants involving bioaugmentation or its ligninolytic enzymes (Torres-Farradá et al., 2024), but till date only two reports have highlighted their potential to produce biosurfactant or emulsifying agent and have established its correlation in degrading them. Song (1999) reported degradation of pyrene using *Phanerochaete chrysosporium* in shaking conditions and suggested that solubilization of pyrene might be due to production of biosurfactant. Similarly, Nikiforova et al. (2009) reported for the first time production of an emulsifying agent after 20 days of cultivation of *Pleurotus ostreatus* during degradation of 2,2′-diphenic, phthalic and α-hydroxy-β-naphthoic acids, fluoranthene, phenanthrene, pyrene, anthracene, fluorene, and chrysene. Muthukumar et al. (2022) has reported that the biosurfactant producing potential of a microbe increases the bioavailability of the hydrophobic pollutants for biodegradation by initially solubilizing them and enhancing their mass transfer rate. In addition to the bioremediation of soil, it is also important that the soil health damaged by the pollutants be restored by replenishing the minerals to protect the plants from oxidative damage. Only few reports have described WRF as plant growth promoting fungus (PGPF) highlighting potential of *Phanerochaete chrysosporium, Trametes versicolor, Lentinus edodes, P. floridensis*, and *P. brevispora* to produce indole acetic acid, gibberellin, and siderophores (Yin et al., 2022; Chandra et al., 2019).

Keeping above parameters in mind, it was necessary to isolate a versatile ligninolytic white rot fungus with multiple traits which can solubilize the structures of benzene phenolic derivatives—bisphenol A/triclosan by producing biosurfactant and concomitantly release certain plant growth promoting (PGP) factors which can relieve the soil from abiotic stress caused due to contaminants. The action of the expressed lignin-modifying enzymes in presence of EDCs has also been correlated with their degradation, and accordingly, a putative degradation pathway has been proposed as per intermediate metabolites identified by GC-MS/LC-MS/MS. The biosurfactant produced during degradation has also been characterized.

## 2 Materials and methods

### 2.1 Chemicals

The chemicals, solvents, and reagents used were of analytical (AR) grade. Bisphenol A (BPA) and triclosan (TCS) were obtained from Tokyo chemical industry (TCI), and ABTS, phenol red, and veratryl alcohol were purchased from sigma Aldrich.

### 2.2 Sampling and isolation

Freshly excised bark samples showing fungal growth were collected from regions of Himachal Pradesh (31.10°N 77.10°E), Uttarakhand (30.45°N 78.25°E), and New Delhi (28.61°N, 77.19°E) (India) and packed in autoclaved polyethylene bags pre-rinsed with 70% ethanol and stored at 4°C.

From the stored bark samples, the isolation of white rot fungus was carried out using tissue culture method. The samples were cut into pieces of suitable size of 1–2 cm and surface-sterilized with 0.1 % HgCl_2_ followed by washing with sterile distilled water and 70 % ethanol for few seconds. Subsequently, pieces of surfaced sterilized samples were placed onto GYMP medium (pH 5) agar plates with composition (g/L)—malt extract (20.0); KH_2_PO_4_ (0.5); MgSO_4._7H_2_O (0.5); Ca(NO_3_)_2_.4H_2_O (0.5); agar (20.0); yeast extract (10.0); peptone (10.0), and glucose (10.0). This medium contained the antifungal agent benzimidazole (0.1%) to prevent filamentous fungi growth and chloramphenicol (0.05 mg/ml) to avoid bacterial contamination. The plates were incubated at 30°C for 7 days. The white rot fungal mat obtained was bored with sterile cork borer of 5 mm and placed onto fresh GYMP plates to obtain pure white rot fungus. Sub-culturing was routinely carried out.

### 2.3 Morphological and molecular identification of isolated white rot fungus

For morphological identification, the isolated fungus was stained using lactophenol cotton blue. For molecular identification, genomic DNA was extracted using the mycelial mat formed after 5 days of incubation. The mat was washed with sterile distilled water to remove medium traces and grounded to paste using extraction buffer [1.4M NaCl, 0.1M Tris HCl (pH 8), 0.02 M EDTA, 20 μl β-mercaptoethanol, 2% cetyl trimethyl ammonium bromide (CTAB)] in mortal pastel. This was then incubated at 65°C for 1 h followed by centrifugation at 3,000 rpm for 5 min at 4°C. The supernatant was collected, and phenol at a ratio of 1:1 was added and kept at RT for 20 min. It was then centrifuged at 7,500 rpm for 10 min at 4°C, and the supernatant was extracted using a mixture containing 300 μl phenol and 300 μl chloroform: isoamyl alcohol (24:1) and incubated at RT for 10 min. This was again centrifuged at 7,500 rpm for 10 min at 4°C. To supernatant thus obtained, 600 μl of chloroform: isoamyl alcohol (24:1) mixture was added, and after 10 min, the mixture was centrifuged at 7,500 rpm for 10 min at 4°C. Equal volume of supernatant, isopropanol, and 60 μl of 3N sodium acetate was added and kept at −20°C for 2 h. It was centrifuged at 12,000 rpm for 20 min at 4°C. The pellet obtained was washed with 70% ethanol, air-dried, suspended in 50 μl of TE buffer (10 mM Tris HCL, pH 8, 1 mM EDTA), and stored at 4°C.

#### 2.3.1 PCR amplification

PCR amplification of 18S rRNA gene was performed using the following specific primers: ITS 1 (5′ TCC GTA GGT GAA CCT GCG G 3′) and ITS 4 (5′ TCC TCC GCT TAT TGA TAT GC 3′) primers. PCR amplification was performed in total volume of 25 μl containing 2.5 μl Taq buffer, 0.5 μl MgCl_2_, 0.5 μl dNTP mix, 0.5 μl Taq DNA polymerase, 1 μl both forward and reverse primer, and 3 μl genomic DNA. The volume was made up to 25 μl by adding 16 μl nuclease-free water. The thermal cycle amplification program was performed on a Bio-Rad PCR system 2,400 (Bio-Rad laboratories, USA) with temperature program as follows: 95°C for 5 min, followed by 35 cycles with each cycle comprising of 30 s of denaturation at 95°C, 30 s of annealing of primers at 55°C, 1 min extension at 72°C, and a final extension for 10 min at 72°C. The PCR amplicon was purified to remove contaminants. The purity and size of each PCR product were examined on 1% agarose gel. The amplified 18S rRNA gene in PCR product was sequenced through Sanger sequencing method in Applied Biosystems 3730xL Genetic Analyzers, USA.

#### 2.3.2 Phylogenetic analysis

CLUSTAL W and neighbor-joining methods were used for multiple sequence alignment and phylogenetic tree construction, respectively. Evolutionary history was inferred using the maximum composite likelihood method. Evolutionary analysis was conducted in Molecular Evolutionary Genetic Analysis software (MEGA7).

### 2.4 Exploration of isolated WRF potential

#### 2.4.1 Lignin-modifying enzymes

The ability of the white rot fungus to produce lignin-modifying enzymes—laccase and peroxidase, was evaluated using 0.01% filter-sterilized substrates, for laccase—tannic acid and guaiacol, and for peroxidases—azure B and phenol red were used. Mycelial disks (5 mm diameter) bored from the peripheral region of actively growing culture agar plates were used as inoculum, and the plates were incubated at 30°C for 10 days. Appearance of dark brown color (tannic acid) and reddish-brown color (guaiacol) indicated the production of laccase enzyme. Hyphal lengths were measured to monitor the growth of isolates. Decolorization of azure B and appearance of pink to reddish color zone for phenol red indicated the production of peroxidases.

#### 2.4.2 Plant growth promoting traits

For restoration of soil health, the potential of WRF was evaluated for the production of different PGP traits—HCN and ammonia production, zinc and phosphate solubilization, ACC deaminase activity, indole acetic acid (IAA), and siderophore production.

For HCN production, WRF was inoculated on malt extract agar plates supplemented with glycine (4%) and covered with lid having a Whatman filter paper no. 1 soaked in picric acid solution (2% Na_2_CO_3_ in 0.5% picric acid) on its inner side. The plates were sealed with parafilm and incubated at 30°C for 7 days and observed for color change from yellow to brown (El-Rahman et al., 2019).

The zinc solubilizing potential was examined via solubilization zone and solubilization index (SI) as per Bhatt and Maheshwari (2020) on Bunt and Rovira media plates supplemented with 0.1% insoluble zinc sources viz., ZnSO_4_ and incubated at 30°C for 7 days. SI was calculated using formula—SI = (colony diameter – halo zone diameter/colony diameter).

For ammonia production, WRF was grown in 4% peptone broth. Following 7 days of incubation, 0.5 ml of Nessler's reagent was added in culture broth and observed for development of brown color indicative of ammonia production and measured at 450 nm (Bhatt and Maheshwari, 2020).

The phosphate solubilizing activity was detected using Pikovskaya's agar plates. Appearance of clear halo zone around the fungal colony after 7 days (30°C) indicated the positive results (Bhattacharyya et al., 2020).

The potential of WRF to produce ACC deaminase enzyme was detected by inoculating one plug of 5 mm diameter in Dworkin and Foster (DF) salt agar medium (4.0 g/l KH_2_PO_4_, 6.0 g/l Na_2_HPO_4_, 0.2 g/l MgSO_4_·7H_2_O, 2.0 g/l glucose, 2.0 g/l gluconic acid and 2.0 g/l citric acid with trace elements: 1 mg/l FeSO_4_.7H_2_O, 10 mg/l H_3_BO_3_, 11.19 mg/l MnSO_4_.H_2_O, 124.6 mg/l ZnSO_4_.7H_2_O, 78.22 mg/l CuSO_4_.5 H_2_O, 10 mg/l MoO_3_, pH 7.2) supplemented with 3.0 mM 1-aminocyclopropane-1-carboxylic acid (ACC) as the only sole nitrogen source. After 7 days of incubation at 30°C, the appearance of growth was considered as positive for ACC deaminase production. For quantitative analysis of ACC deaminase activity, the fungus was grown in DF medium amended with 3 mM ACC at 30°C, 150 rpm for 48 h, and then centrifuged at 10,000 rpm, 4°C for 10 min. The liberation of α-ketobutyrate in the supernatant after ACC degradation was read at 540 nm using α-ketobutyrate standard curve (Murali et al., 2021).

For indole acetic acid (IAA) producing potential, the WRF was grown in minimal medium supplemented with 100 μg ml/L tryptophan at 30°C for 7 days and then vigorously mixing the culture filtrate (1 ml) with 4 ml of Salkowski's reagent (50 ml, 35% perchloric acid and 0.5 M FeCl_3_). Appearance of pink color after 30 min incubation, indicative of IAA production, was quantified at 520 nm using UV-Vis spectrophotometer (UV-1900i, Shimadzu), and the concentration was determined using IAA standard curve.

Siderophore production by WRF was studied using chrome azurol sulfonate (CAS) agar medium. Appearance of orange halo zone around the fungal colony reflects positive result due to the chelation of iron from CAS by the siderophore (Bhattacharyya et al., 2020). Quantitatively, siderophore production was performed using CAS-shuttle assay at 630 nm and estimated as % siderophore unit = [Ar–As)/Ar] × 100, where Ar = absorbance of reference (minimal medium + CAS assay solution), and As = absorbance of the sample.

#### 2.4.3 EDC tolerance

The EDC tolerance at 10, 50, and 100 ppm was analyzed for isolated white rot fungus toward bisphenol A (BPA) and triclosan by placing a 5 mm diameter mycelial plug from a 5- to 7-day-old culture on 90 mm GYMP agar plate supplemented with respective EDCs concentration. Plates with no EDCs served as control. After 10 days of incubation at 30°C, growth in terms of radial extension of the mycelium was recorded.

### 2.5 Optimization with respect to degradation of EDC—bisphenol A/triclosan

The degradation of EDCs under shake flask conditions was determined by inoculating five active mycelial agar plugs of 5 mm diameter in 100 ml GYMP medium (pH 5) containing 50 ppm of BPA and triclosan. The pH of the medium was taken as pH 5 as many reports have highlighted the degradation of BPA/TCS in optimum pH range of 5–6 (Hongyan et al., 2019). Two modes were adopted—in mode A, EDC either BPA or TCS was added at 0 day and mode B where individual EDC was added after 5 days of prior growth. All the flasks were incubated at 30°C, 150 rpm for 15 days. Aliquots were withdrawn at 3-day interval, centrifuged at 3,000 rpm for 10 min, and examined for pH change, extracellular lignin-modifying enzymes—laccase and peroxidases, residual EDC content, and their metabolites. The dry weight (g/ml) of fungal biomass was also determined. In addition to this, the comparative effect of static condition against the shaking in degradation was monitored for mode B and the thick fungal mat formed under static conditions was evaluated for EDC bioaccumulation.

#### 2.5.1 Correlation of EDC degradation with biosurfactant production

The biosurfactant producing potential of WRF *Hypocrea lixii* was evaluated in the presence and absence of EDCs quantitatively by measuring surface tension and emulsification index EI (%) (Joy et al., 2017). The surface tension of the culture filtrate was measured using digital surface tensiometer (SEO, Instruments, Korea) working on principle of Du Nouy ring method. Lowering of surface tension values with respect to uninoculated control flask is indicative of production of biosurfactant.

#### 2.5.2 Characterization of biosurfactant

The biosurfactant produced in the culture filtrate during degradation was extracted by acidifying culture filtrate with 6N HCl to pH 2.0. The suspension was kept overnight for precipitation at 4°C and then centrifuged. The precipitate obtained was extracted twice with chloroform: methanol (2:1 v/v) mixture. The extracts were pooled and concentrated under a vacuum using a rotary evaporator. A TLC spotted with standards and concentrated partially purified samples was run in a solvent system comprising of chloroform/methanol/water (65:15:2, v/v/v). For glycolipids, the TLC was sprayed using orcinol-sulfuric acid reagent (1% in conc. sulfuric acid) and heated to 110°C. If brown spots appear, it indicates the presence of glycolipids. On the other hand, if on spraying ninhydrin reagent (0.2% ninhydrin in ethanol) red-pink spots appear, it revealed the presence of lipopeptide BS (Muthukumar et al., 2023; Joy et al., 2017). Further characterization was accomplished by Fourier transform infrared spectroscopy (FTIR) analysis in the range between 400 and 4,000 cm^−1^. For GC-MS, the partially purified biosurfactant (10 mg) was dissolved and derivatized in 1 mL of 1 M HCl-methanol in a closed screw-cap tube at 100°C for 4 h. The derivatized product containing the fatty acid methyl esters (FAMEs) was partitioned by adding double distilled H_2_O (1 mL), and the organic phase was extracted over anhydrous sodium sulfate for moisture removal. Approx. 1 μl of the sample was injected into TQ8040 Shimadzu GC-MS equipped with RTX- 5MS fused silica capillary (2.5 μm thickness, 2.5 mm internal diameter, 30 m length). The carrier gas consisted of helium at a flow rate of 1 ml/min. The operating temperatures of the injector and column were 260°C and 140°C. Electron impact mass spectra were recorded at 70 KeV. For characterization using LC-ESI-MS/MS triple quadrupole mass spectrometer (Shimadzu 8050), a prep TLC was run. The silica was scraped and vortexed in methanol to elute the sample. Finally, the sample was centrifuged and the supernatant approx. 1 μl was loaded onto C18 reverse phase column (2.1 × 150 mm). The separation was achieved using the mobile phase (A) 5 mM ammonium formate, 0.1% formic acid in water and (B) 5 mM of ammonium formate, 0.1% formic acid in methanol. The flow rate was maintained at 0.4 ml/min following a gradient of 5% methanol for 1 min and 50% methanol for 1–2 min. The eluate was directed to the mass spectrometer through electrospray ionization source operated in positive mode, and the mass spectra were recorded within the mass range of 300–1,500 m/z. Detection was performed in following conditions of ion source and mass spectrometer: nebulizing gas flow rate 3 L/min, heating gas flow rate 10 L/min, interface temperature 300°C, DL temp. 250°C, and drying gas flow rate 10 L/min.

#### 2.5.3 Correlation of EDC degradation with enzymes

To elucidate the enzyme system involved in the degradation of EDCs, the role of extracellular and intracellular enzymes was monitored.

##### 2.5.3.1 Extracellular lignin-modifying enzymes assay

Lignin-modifying enzymes—laccase and peroxidases—were quantitatively analyzed by spectrophotometer (UV-1900i- Shimadzu) in triplicates.

**Laccase:** Spectrophotometric determination of laccase was performed according to Rodríguez-Delgado et al. (2016). The enzyme activity was assayed using 5 mM ABTS as substrate in McIlvain buffer (0.2 M sodium phosphate dibasic/citric acid 0.1 M), pH 3. The increase in absorbance was read at 420 nm (ε_420_36 000 M^−1^ cm^−1^). The enzyme activities were expressed in U/L. One-unit activity was defined as the amount of laccase required to oxidize 1 μmol of ABTS per ml per min.

**Manganese peroxidase:** Spectrophotometric analysis of manganese peroxidase activity was performed according to Kuwahara et al. (1984). The reaction mixture contained 0.2 ml of 0.5% bovine albumin serum, 0.05 ml of 2 mM MnSO_4_, 0.1 ml of 0.25 M sodium lactate, 0.1 ml of 0.1% phenol red, 0.5 ml enzyme, and 0.05 ml of 2 mM H_2_O_2_ in 0.2 M sodium succinate buffer (pH 4.5). The reaction was stopped using 0.04 ml sodium hydroxide (2 N). The increase in absorbance was read at 610 nm (ε_610_22000 M^−1^ cm^−1^), and the activity was expressed in U/L. One-unit activity was defined as the amount of manganese peroxidase required to oxidize 1 μmol of phenol red per ml per min under standard assay conditions.

**Lignin peroxidase:** Using veratryl alcohol as the oxidation substrate, the activity of lignin peroxidase enzyme was determined spectrophotometrically by observing an increase in absorbance at 310 nm (ε_310_9300 M^−1^ cm^−1^) (Arora and Gill, 2001). The reaction mixture contained 250 μl veratryl alcohol (10 mM), 500 μl sodium tartrate buffer (125 mM, pH 3.0), 250 μl enzyme extract, and 250 μl hydrogen peroxide solution (2 mM). One unit of enzyme activity was defined as the amount of enzyme which could produce 1 mol of veratryl aldehyde by the oxidation of veratryl alcohol per ml per min.

##### 2.5.3.2 Intracellular cytochrome P450 assay

To understand the role of intracellular cytochrome P450 monooxygenase (Cyt P450), inhibition of Cyt P450 was conducted in mode B set by adding 1.0 mM of 1-aminobenzotriazole (ABT) at the start of the cultivation in GYMP medium containing 50 ppm EDC (Da Silva Coelho-Moreira et al., 2018) and incubating flasks for 15 days. The metabolites if formed were analyzed after extraction.

### 2.6 Extraction of residual EDCs

For residual EDC and their metabolites formed during degradation, the culture filtrates were extracted with dichloromethane (DCM) in the ratio 1:1. The organic layer was dried and resuspended in methanol: water (1:1) for quantifying spectrophotometrically or by GC-MS for BPA and LC-MS/MS for triclosan.

#### 2.6.1 Spectrophotometric analysis of residual EDCs

Spectrophotometrically, residual EDCs in the extracted samples dissolved in methanol: water were analyzed using UV-Vis spectrophotometer (UV-1900i, Shimadzu) at 275 nm for BPA and 250 nm for triclosan (Mohd Khori et al., 2018; de Lima et al., 2021). Standard curve of each EDC was prepared in the range of 5–50 μg/ml from the stock solution dissolved in methanol with blanks containing no EDC. Triplicates assays were conducted. The percentage degradation of EDCs was calculated as follows:


(1) $$\begin{array}{l} \text{\%degradation}=\frac{I_{c\;-\;F_{c}}}{I_{c}}\;\times 100 \end{array}$$

where I_C_ = initial EDC concentration, and F_C_ = final EDC concentration.

#### 2.6.2 Mass spectrometric analysis of residual BPA and triclosan and their metabolites

##### 2.6.2.1 GC-MS analysis of residual BPA

GC-MS analysis of the control sample (only BPA) and the test samples were carried out in duplicates using Agilent Technologies 5975C inert XL EI MSD. The ionization voltage was 70eV. Gas chromatography was conducted in the temperature programming mode with a column DB−5 (30 m × 250 μm × 0.25 μm). The initial column temperature was 50°C for 2 min, then increased linearly at 8°C per min up to 260°C, and held for 7 min. The GC–MS interface was maintained at 280°C. Helium was used as a carrier gas at a flow rate of 1.2 ml/min with 38 min run time. Based on the m/z presented in the mass spectrum, the possible structure of the intermediate products was identified.

##### 2.6.2.2 LC- MS/MS analysis of residual triclosan

The LC-MS/MS analysis of the control sample (only TCS) and test samples were carried out in duplicates carried out using Shimadzu 8050 connected with ESI triple quadrupole mass spectrophotometer. Approx. 2 μl was injected into C18 with dimension 2.1 × 150 mm. The mobile phase of 5 mM of ammonium acetate in water and acetonitrile at a flow rate of 0.4 ml/min was used. The analytical gradient used for analysis of triclosan was as follows from 65% to 35% of acetonitrile in 7 min, 100% of acetonitrile for 2 min, and 35 % of acetonitrile in 3 min. Chromatographic column effluent was directed to the mass spectrophotometer through electrospray ionization source. The source was operated in negative mode. Detection was performed in following conditions of ion source and mass spectrometer: nebulizing gas flow 3 L/min, heating gas flow 10 L/min, interface temperature 300°C, DL temp. 250°C, and drying gas flow 10 L/min. The m/z in the mass spectrum was used to elucidate the possible structure of the intermediate products.

### 2.7 Bioaccumulation

The thick fungal mat obtained during static cultivation in mode B set (EDC added after 5 days of prior growth) was used for bioaccumulation study. After 15 days of growth, the mycelia were filtered using pre-weighed Whatman filter paper no. 1, washed 2–5 times with sterile distilled water, and crushed in chilled 10% methanol using mortal pastel. The mixture was incubated at 4°C for 2 h followed by centrifugation at 5,000 rpm, 4°C for 10 min. Absorbance was then read at 275 nm for BPA and 250 nm for triclosan, respectively, to determine the bioaccumulated EDC content.

#### 2.7.1 Scanning electron microscopy analysis

The bioaccumulated EDC content on fungal surface was further examined using SEM [Evo 18 special edition (Zeiss)] under vacuum at 20 KV. The dry fungal biomass was washed twice with phosphate buffer saline solution (PBS) and then used for SEM analysis along with control. Both control and EDC absorbed fungal biomass were mounted on stainless steel with coating of thin layer of gold under vacuum. Energy dispersive X-ray (EDX) analysis was performed to confirm the elemental content within fungal biomass (control and EDCs adsorbed).

## 3 Results and discussion

### 3.1 Morphological and molecular identification of isolated white rot fungus

**Figure 1:**
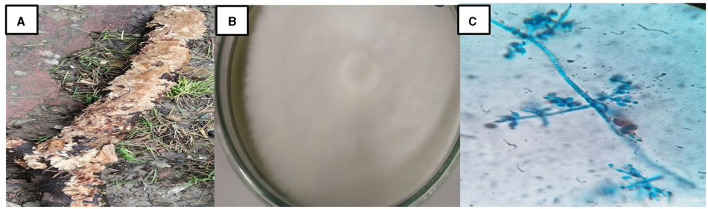
*Hypocrea lixii*. **(A)** Fruiting body. **(B)** Pure culture. **(C)** Microscopic view.

**Figure 2:**
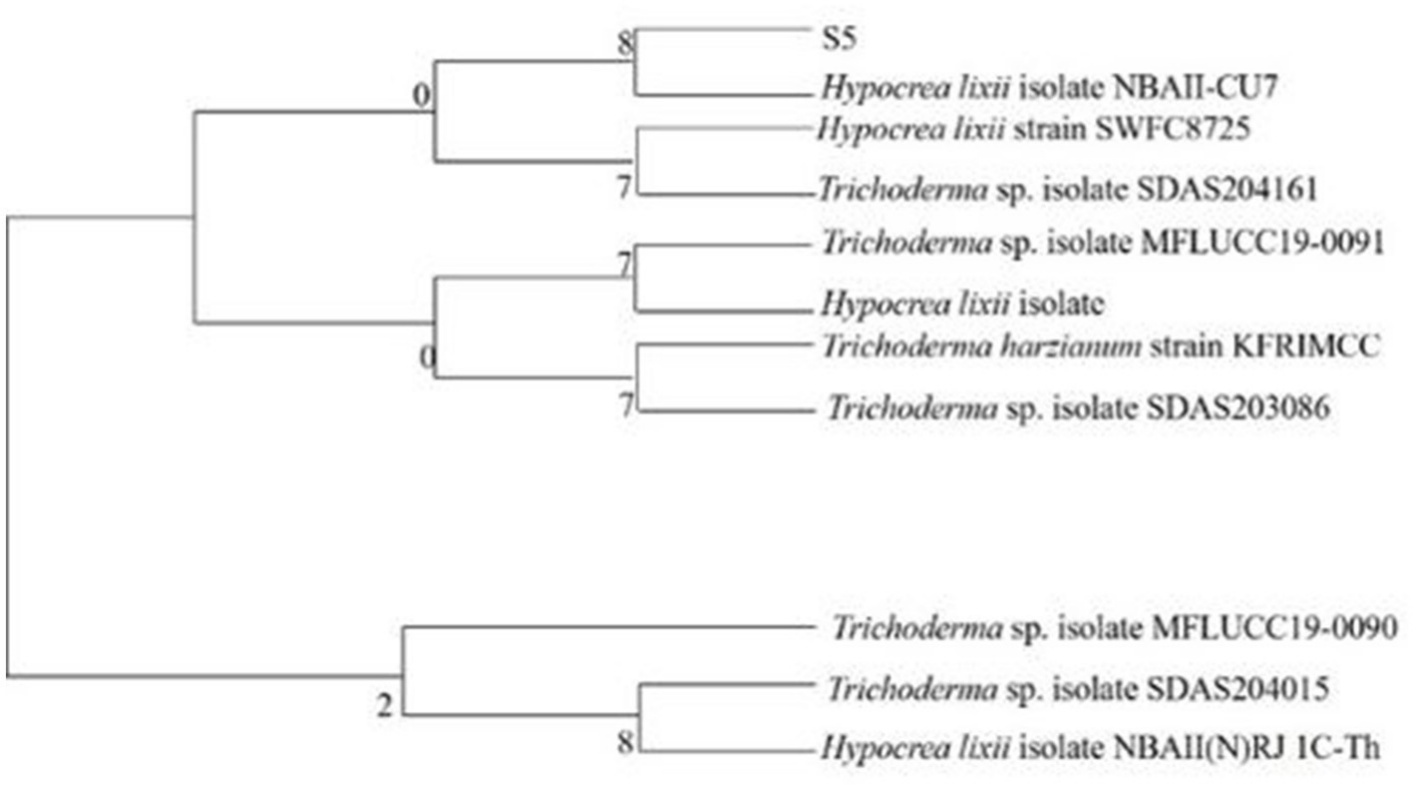
Phylogenetic analysis of *Hypocrea lixii*.

The isolated fungus (S5) grew as white cottony mass on PDA with floccose aerial mycelium. Microscopically, sparingly branched conidiophores were observed with cylindrical to subulate phialides bearing globose or subglobose conidia (Figure 1). The observations suggested it to be *Hypocrea lixii*, the teleomorph state of *Trichoderma harzianum*, and is same as *Trichoderma lixii* as per Index Fungorum (Bohacz and Korniłłowicz-Kowalska, 2020). He et al. (2022) and Shi et al. (2021) concluded *Hypocrea* genus under white rot fungi as it causes wood decay. The 18s rRNA gene sequence analysis showed 97.30% similarity of S5 with *Hypocrea lixii* NBAII-CU7. The 18S rRNA sequence of isolate from this study has been submitted to the NCBI GenBank database, and the accession numbers (PP741816) have been provided for the same. The phylogenetic analysis is presented in Figure 2.

### 3.2 Plant growth promoting traits

*Hypocrea lixii* showed qualitative positive results for Zn solubilization with SI–3 cm, ACC deaminase activity with growth diameter of 5 cm on ACC (sole nitrogen) supplemented agar plates and for siderophore production with orange halo zone of 4 cm around the mycelial growth. Quantitatively, production of 1.10 μmol/ml ammonia, 5.34 μg/ml IAA, 0.55 mM of ACC, and 76.10% siderophore was recorded. WRF, however, showed negative results for HCN and phosphate solubilization. Yin et al. (2022) also reported release of Fe-binding ligands (71%), indole acetic acid (2.0 μg/ml), and gibberellin (122 μg/ml) in the culture broth in 120 h when inoculated by white rot fungus *Ceriporia lacerata* HG201. Chandra et al. (2019) reported *P. brevispora* to produce IAA with maximum 31 μg/ml in complex (yeast extract glucose) medium followed by *P. floridensis* and *P. chrysosporium* than synthetic (Czapek's Dox) medium.

### 3.3 Lignin-modifying enzymes and EDC tolerance

*Hypocrea lixii* showed positive results for laccase with appearance of dark brown zone of diameter 5 cm on tannic acid agar plates and reddish-brown zone of diameter 2 cm on guaiacol agar plates. Peroxidase production was indicative by the appearance of reddish pink color zone and decolorization zone of diameter 5 cm each on phenol red and azure B agar plates, respectively.

The WRF was observed to be tolerant to EDCs with increase in hyphal growth from 10 to 100 ppm in BPA as 3 cm (10 ppm), 3.5 cm (50 ppm), and 4 cm (100 ppm) and in triclosan as 0.3 cm (10 ppm), 1.2 (50 ppm), and 1.5 cm (100 ppm), respectively (Supplementary Figure S1).

### 3.4 Optimization and correlation of EDC degradation with biosurfactant production

**Figure 3:**
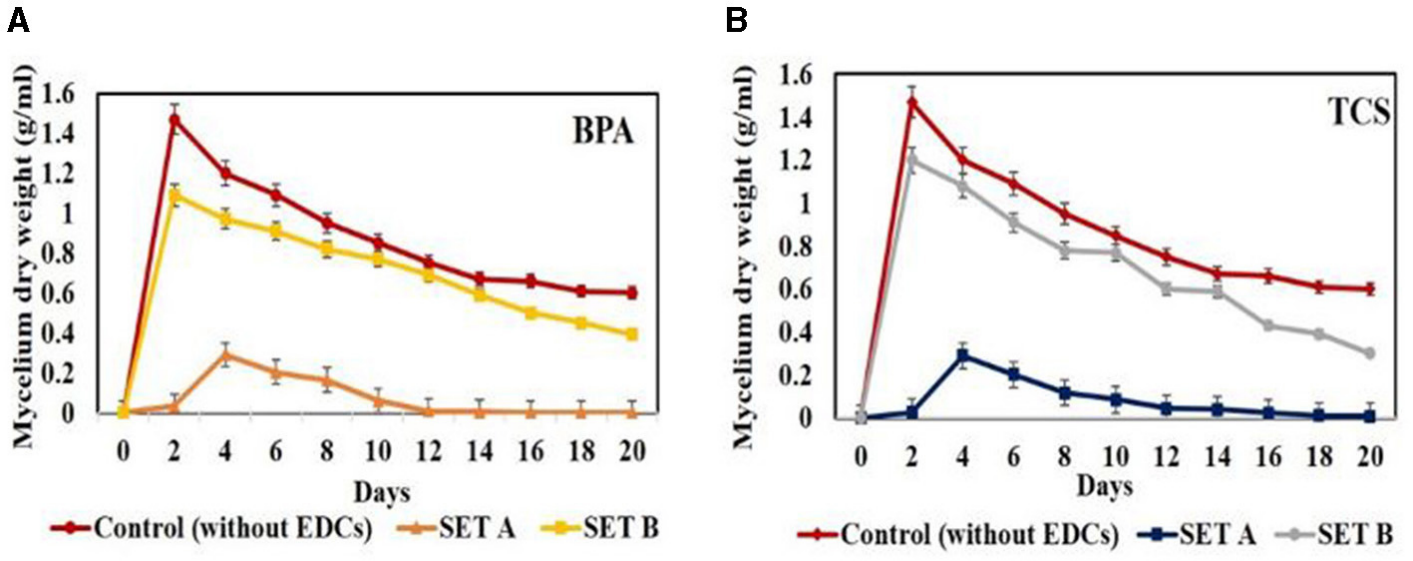
Comparison of growth profile of *Hypocrea lixii* at 30°C, 150 rpm in presence of 50 ppm BPA/TCS in sets **(A, B)** [**(A)**—EDCs added at 0 day of inoculation; **(B)**—EDCs added after 5 days of inoculation] against control set—without EDCs.

The degradation studies in mode A involving addition of EDC (50 ppm) at 0 day revealed on 20^th^ day a drastic growth inhibition of 84% in case of BPA and 97% in case of triclosan, as compared to control (without EDCs). In mode B, wherein individual EDC (50 ppm) was added after 5 days of prior growth, 28% and 14.89% inhibition in growth was observed on 20^th^ day for BPA and triclosan, respectively, against the control (Figure 3). Jasińska et al. (2021) also reported slight inhibition in growth of *M. roridum IM* 6482 in the presence of 50 mg/L BPA in initial hours of cultivation with 3.5 g/L biomass against the control with 4.8 g/L. The growth in later stages was supported by the cometabolites formed after the transformation of EDCs which acted as source of energy and reducing equivalents. Similar to growth inhibition pattern, degradation was also affected with 60.35% degradation of BPA in mode A compared to 78.62% degradation in mode B. In addition, triclosan degradation was observed to be 48.59% in mode A while 69.18% in mode B. This difference in degradation in modes A and B might be due to the disruption of membrane integrity, permeability, and membrane fluidity of cellular structures of white rot fungal inoculum in the presence of EDC in mode A at 0 day of inoculation. The well grown 5-day culture (set B), however, was able to withstand the EDC concentration because of the detoxifying effect of sufficiently produced extracellular oxidative ligninolytic enzymes (Asif et al., 2017). Contradictory to our results, Shin et al. (2007) reported maximum degradation (100%) of BPA (50 ppm) by WRF *Irpex lacteus* on 0-day incubation while slightly lower degradation of 98% when BPA was added in 5-day pre-incubated fungal culture and related it to the exhaustion of growth substrate in 5-day pre-incubated culture (Shin et al., 2007). Under shake flask conditions, better degradation percentage of BPA and TCS was observed in all concentrations (10, 50, and 100 ppm) compared to static conditions. This might be due to improvement in mass transfer with respect to oxygen and uptake of nutrients/xenobiotic compounds as cometabolites from the culture medium, promoting fungal growth and release of oxidative enzymes (Kapoor et al., 2021). The 20^th^ day results showed degradation of BPA at 10 ppm (97.58%), 50 ppm (78.62 %), and 100 ppm (81.5%), respectively. Triclosan also showed maximum degradation on 20^th^ day at 10 ppm (66.54%), 50 ppm (69.18%), and 100 ppm (70.2%), respectively.

**Figure 4:**
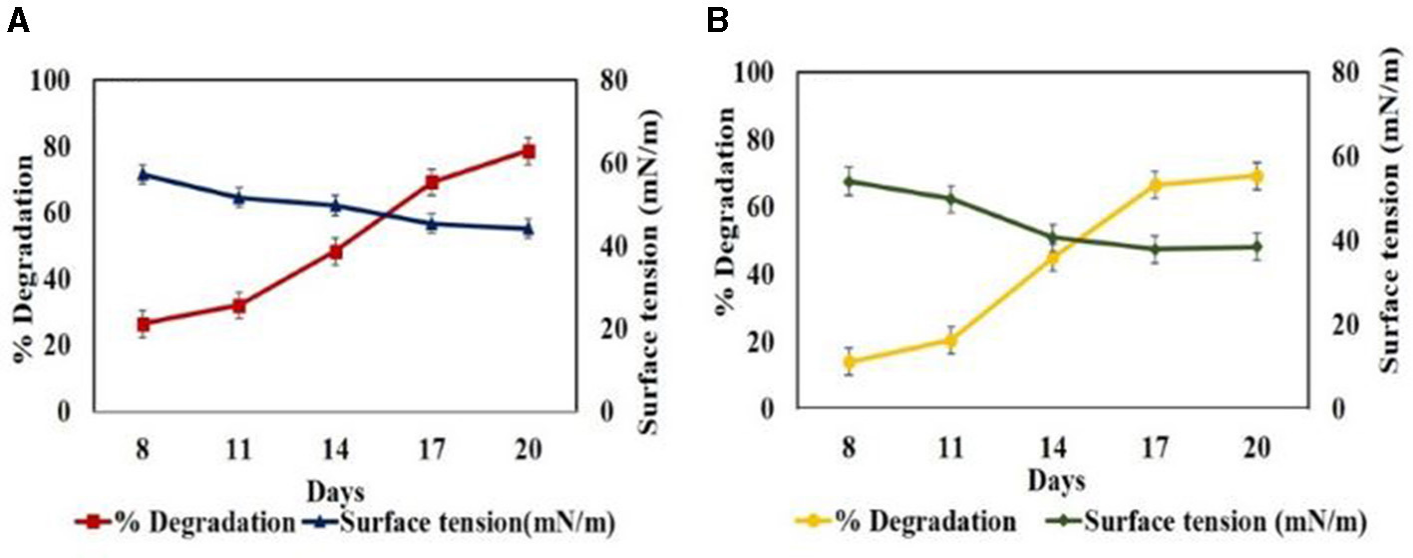
Time profile biosurfactant production by *Hypocrea lixii* during degradation of 50 ppm EDC in set B at pH 5, 30°C, 150 rpm. **(A)** BPA and **(B)** TCS.

Furthermore, in set B during the degradation profiling studies of BPA (50ppm) under shake flask conditions, a decrease in surface tension values of the culture filtrate was observed from 57.02 mN/m in 8^th^ day to 44.16 mN/m with EI-−29.41% in 20^th^ day against the uninoculated control with EDCs (59.05 mN/m). Similarly, during degradation of 50 ppm TCS, surface tension values decreased from 54.02 to 38.49 mN/m with EI of 23% on 20^th^ day. In both cases, the decrease in surface tension values from 0^th^ day to 20^th^ day correlated with the increase in degradation from 26.4% to 78.62% for BPA and 14% to 69.18% for TCS as evident from Figure 4. This can be associated with the production of BS in the culture medium which mobilizes, solubilizes, and emulsifies the hydrophobic EDCs, increasing their bioavailability to fungus and hence their degradation and uptake as carbon and energy source (Patowary et al., 2022). Only very few fungal species of *Trichoderma, Penicillium, Fusarium, Mucor*, and *Aspergillus* have been reported to produce BS (Maamar et al., 2020). Bezza and Chirwa (2016) reported the enhancement of degradation of PAHs up to 86.5% in the presence of lipopeptide BS compared to that in the absence of BS with only 57% suggesting emulsification of PAHs by BS followed by degradation. Nikiforova et al. (2009) also reported the increase in emulsifying activity of cultivation medium in the presence of PAHs from 2 to 48 times and stated that emulsifying activity depends inversely on the water solubility of PAHs. Femina et al. (2021) have reported that lipopeptide BS because of their versatile properties have the potential to remediate organic and inorganic pollutants.

### 3.5 Characterization of biosurfactant

**Figure 5:**
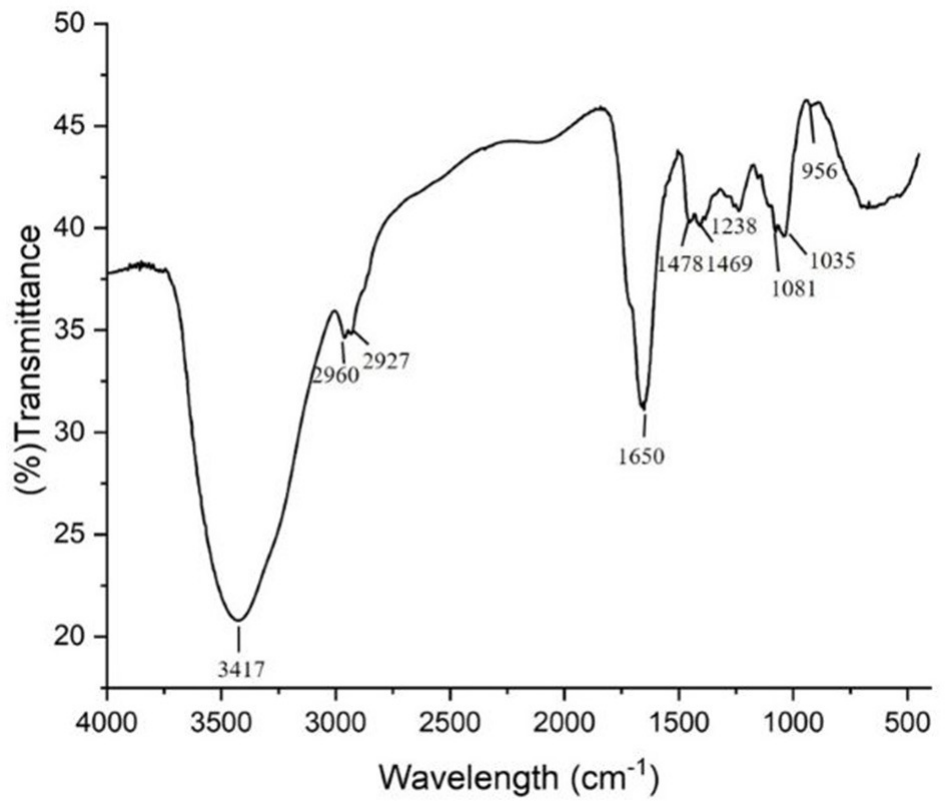
FTIR spectrum of biosurfactant produced by *Hypocrea lixii*.

During EDC degradation via WRF *Hypocrea lixii*, the biosurfactant (BS) produced was found to be of lipopeptide nature as ninhydrin reagent showed development of two prominent pink spots at Rf value of 0.7 and 0.12 on TLC sheet. The FTIR spectrum of the biosurfactant (Figure 5) revealed the presence of carboxylic functional groups and aliphatic amines with a peak between 3,000 and 3,600 cm^−1^, representing -OH and N-H stretching. The peaks at 2,960 cm^−1^ and 2,927 cm^−1^ represented C-H stretching, which might be due to the –CH stretch arising from the amino acids as well as the aliphatic chain or alkyl part of the lipopeptide. The spectral peaks at 1,650 cm^−1^ and 1,238 cm^−1^ showed an amide or peptide bond formed by C-O stretching vibrations and is specific for lipopeptide type of biosurfactant. The peaks at 1,035 cm^−1^ and 956 cm^−1^ indicated the presence of C–N aliphatic amines. Peaks at 1,478 cm^−1^ and 1,469 cm^−1^ indicated C-H bending vibrations commonly observed in compounds with aliphatic chain. Similar spectrum confirming lipopeptide type of biosurfactant has been reported by Rasiya and Sebastian (2021) and Umar et al. (2021). The fatty acid profile of the biosurfactant obtained from GC-MS revealed the presence of tridecanoic acid (C_13_), tetradecanoic acid (C_14_), pentadecanoic acid (C_15_), hexadecenoic acid (C_16_), octadecanoic acid (C_18_), and nonadecanoic acid (C_19_) and showed similarity to the fatty acid profile of surfactin standard as evident in Table 1. The LC-ESI-MS/MS profile showed the presence of 680 m/z, 702 m/z, 1,022 m/z, and 1,044 m/z ions in the positive mode (Supplementary Figure S2). The chromatogram peak with 1,022 m/z and 1,044 m/z is attributed to two isomers of surfactin. The presence of fragment ion peak at m/z 680 may be of surfactin isoform as per Ma et al. (2016) who reported that the common peaks of surfactin isoform ion occur at *m/z* 685, 671, or 681. Gurjar and Sengupta (2015) also stated that the peak at 685.7 m/z strongly corresponds to peptide moiety from amino acid (AA) 2 to AA7 after preferential opening of the lipopeptide molecule ring at the ester site followed by fragmentation between AA1 and AA2.

**Table 1:** Composition of fatty acids determined by GC-MS.

**Fatty acid composition**	**Relative area percentage**	
**Lipopeptide**	**Surfactin (sigma)**	**Biosurfactant from** ***Hypocrea lixii***
Tridecanoic acid (C13.0)	-	3.49
Tetradecanoic acid (C14.0)	3.30	9.59
Pentadecanoic acid (C15.0)	37.75	2.71
Hexadecenoic acid (C16.0)	34.60	53.91
Octadecanoic acid (C18.0)	23.12	32.72
Nonadecanoic acid (C19.0)	1.21	-

### 3.6 Correlation of degradation of EDC with enzymes

#### 3.6.1 Role of extracellular lignin-modifying enzymes

**Figure 6:**
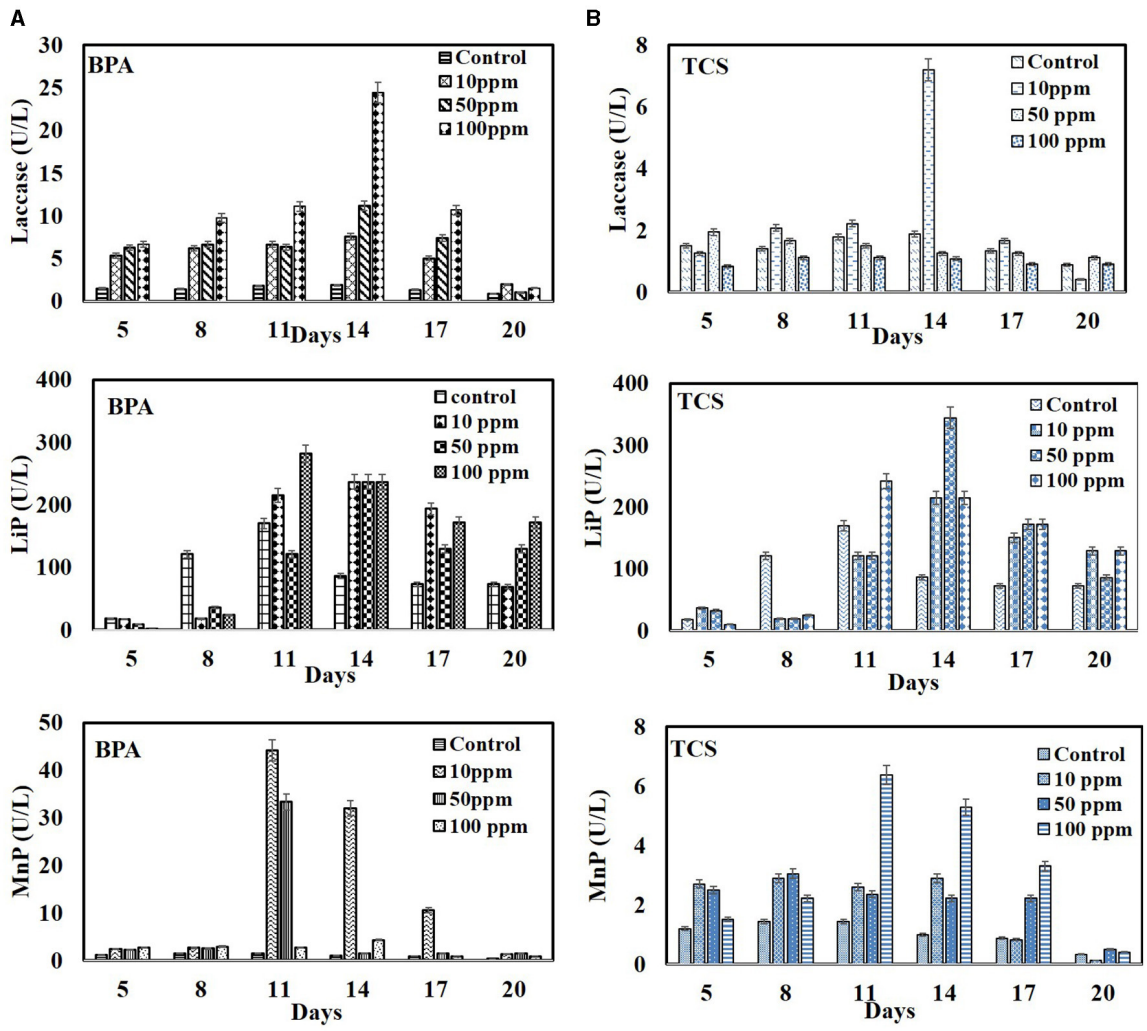
Time profile production of lignin-modifying enzymes (laccases, MnP, and LiP) by *Hypocrea lixii* during degradation of EDC at 10, 50, and 100 ppm. **(A)** BPA and **(B)** triclosan.

The degradation pattern of EDCs in set B showed the induced synergistic expression of LMEs with the highest LiP being 281.69 U/L in 100 ppm BPA on 11^th^ day and 344 U/L in 50 ppm triclosan on 14^th^ day as compared to controls (without EDC) with 170.06 U/L and 86.55 U/L on 11^th^ and 14^th^ day, respectively. BPA also induced laccase activity with maximum in 100 ppm (24.44 U/L) as compared to control with 1.88 U/L. Manganese peroxidase expression was high at low BPA concentration of 10 ppm (44.16 U/L) as compared to control with 1.44 U/L on 11^th^ day (Figure 6A). Loffredo et al. (2013) also reported the stimulated expression of ligninolytic enzymes with increase in BPA concentration and correspondingly its high removal efficiency using fungi *T. versicolor, S. hirsutum*, and *P. ostreatus*. Enhancement of all the three lignin-modifying enzymes in the presence of BPA without inhibiting fungal growth have also been reported by Zeng et al. (2017) and Takamiya et al. (2008).

The activities of laccase and MnP were, however, inhibited at higher TCS concentrations with maximum activity of laccase (7.2 U/L) and MnP (6.52 U/L) in 10 ppm (Figure 6B). Mallak et al. (2020) also reported TCS concentration-dependent response of two white rot fungi—*T. versicolor* and *P. ostreatus—*in enhancement of laccase and manganese peroxidase activities. Furthermore, Cajthaml et al. (2009) reported that inhibition in enzyme activity might be due to the suppressive effect imposed by the accumulated oxidation products. The induced expression of LiP and laccase and the corresponding enhanced degradation of BPA/TCS may also be supported by the lipopeptide biosurfactant produced by the WRF. Several reports have highlighted the improvement in degradation efficiency of xenobiotic pollutants because of the stimulating effect of biosurfactants in enhancing the activities of enzymes. Tang et al. (2005) reported that rhamnolipid BS increased the titers of lignin peroxidase and Mn peroxidase by *Phanerochaete chrysosporium* and lignin peroxidase and laccase *by Penicillium simplicissimum*. Zhou et al. (2011) reported that tea saponin, a biosurfactant, mitigates the negative effects of phenol on fungal growth and consequently improves laccase production and phenol degradation. It has been reported that the interactions which play a major role in changing the confirmation of enzyme during degradation are the electrostatic interactions between the surfactant head group and the charged amino acid residues of the enzyme and the hydrophobic interactions between the alkyl chains of the surfactant and the hydrophobic amino acid residues of the enzyme. These interactions depend on the characteristics of the enzyme and the surfactant, such as the charge and size of head group, the length of the surfactant, and the hydrophobicity of its alkyl chain (Aguirre-Ramírez et al., 2021).

#### 3.6.2 Role of intracellular cytochrome P450 enzyme

The addition of 1-aminobenzotriazole (ABT), an intracellular enzyme Cyt P450 inhibitor, revealed no significant change in degradation pattern of 50 ppm of bisphenol A/triclosan suggesting sole role of LMEs. Mir-Tutusaus et al. (2014) reported the important role of cytochrome P450 in degradation of three pollutants—imiprothrin, (IP), oxytetracycline (OTC), and carbofuran (CBF) by WRF *Trametes versicolor* but not in degradation of cypermethrin (CP).

### 3.7 Changes in pH during biodegradation studies

The variation in pH from the initial medium pH 5, monitored during the degradation of BPA and TCS, revealed a slight increase in the pH value in the beginning for both the EDCs followed by a decrease to pH 4.5 and 4.8 for BPA and TCS, respectively (Supplementary Figure S3). This lowering of pH might be due to the breakdown of BPA/TCS into slight acidic metabolites as also reported by Elumalai et al. (2021) during the biodegradation of hydrophobic organic pollutants.

### 3.8 Identification of metabolites by mass spectroscopic studies

**Figure 7:**
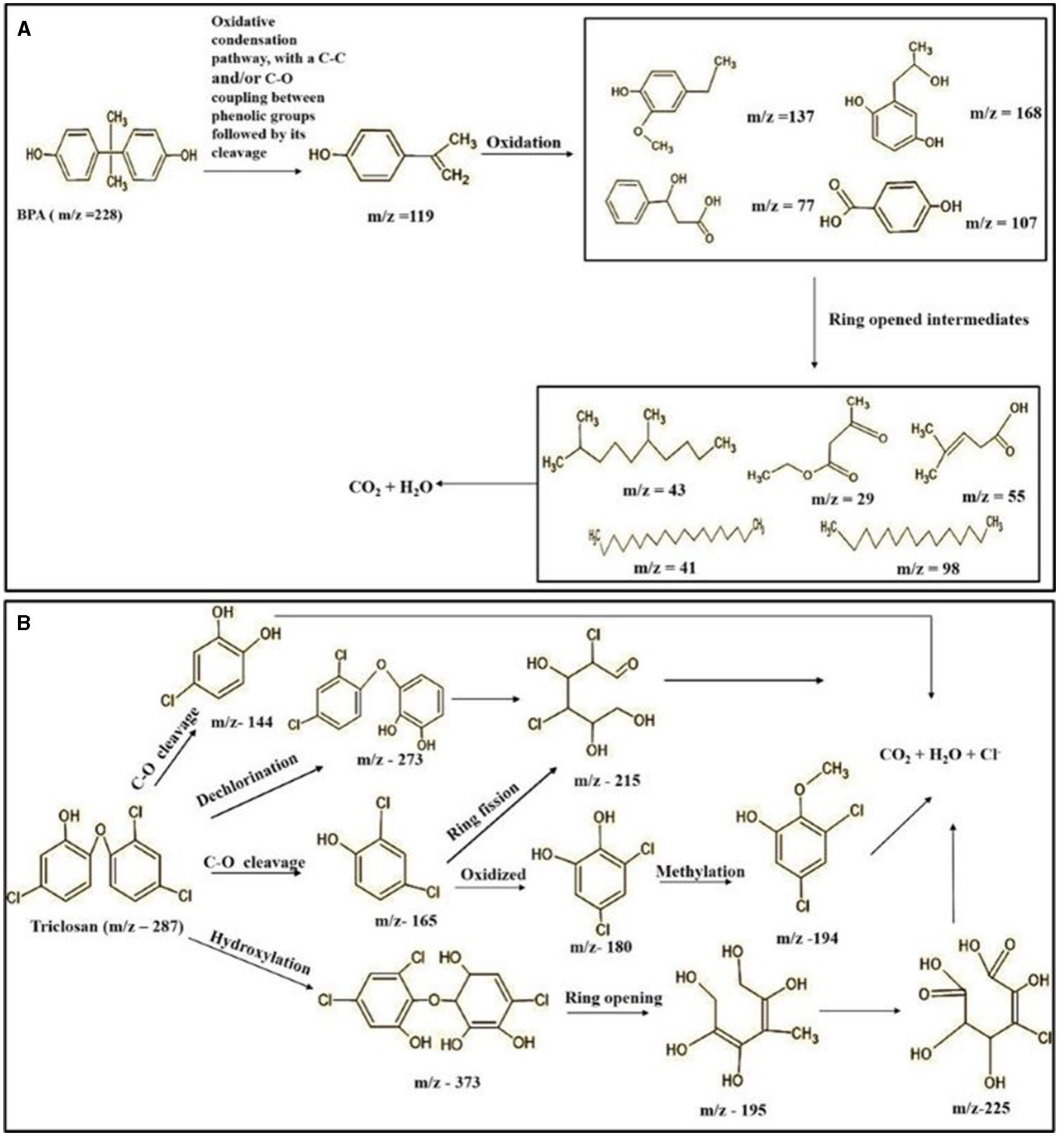
Possible degradation pathways of EDC in synthetic medium by *H. lixii* at pH 5, 30°C. **(A)** BPA and **(B)** triclosan.

The GC-MS analysis of 11^th^ day degradation sample of BPA (50 ppm) showed appearance of eleven metabolites, with further less number on 14th day and finally no emergent peak on 20^th^ day (Supplementary Figures S4–S7; Table 2). Thus, a putative degradation pathway of BPA was proposed (Figure 7A) which showed oxidative condensation forming oligomers with C–C and/or C–O bonds between phenolic groups followed by its cleavage forming p-isopropenylphenol with m/z =119. Further successive oxidation of p-isopropenylphenol led to formation of acidic metabolite [3-hydroxy-3-phenyl propionic acid (m/z = 77)] and hydroxylated metabolites [4-ethyl-2-methoxyphenol (m/z = 137), 4 hydroxy benzoic acid (m/z = 107), and 2-(2-hydroxypropyl)-benzene1, 4-diol (m/z = 168)]. These metabolites are related to the enzymatic actions of extracellular enzymes as laccase and peroxidase (Mtiba et al., 2018). These intermediates could have been sequentially oxidized via laccase through ring rupturing reactions to yield aliphatic compounds as nonadecane (m/z = 41) and hexadecane (m/z = 98) which are known to be integrated into cellular metabolism of fungi.

**Table 2:** Identification of intermediate metabolites of bisphenol A by GC-MS and triclosan by LC-MS/MS formed during degradation by *H. lixii*.

**S. no**	**m/z**	**Metabolites**	**Structure**
**GC-MS analysis of BPA**			
1	228	BPA	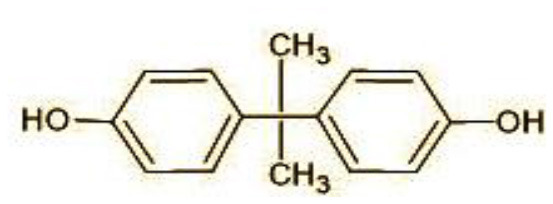
2	29	Ethyl-3-oxo-butanoate (C_6_H_10_O)		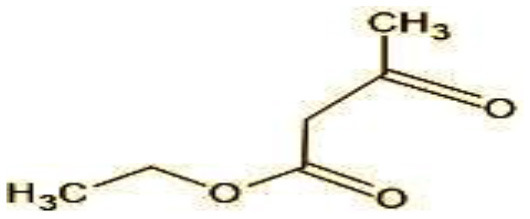
3	41	Nonadecane (C_19_H_40_)			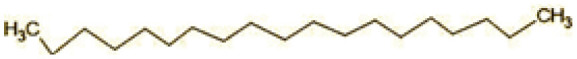
4	43	2,6-Dimethyldecane (C_12_H_26_)				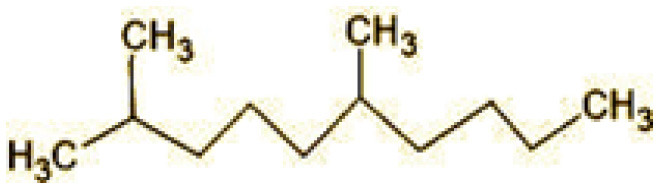
5	55	4 Methylpent-3-oic acid (C_6_H_10_O_2_)	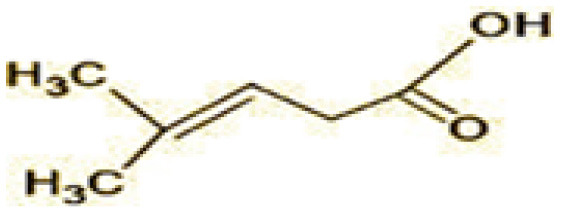			
6	59	Ethyl-3-ethoxypropanoate		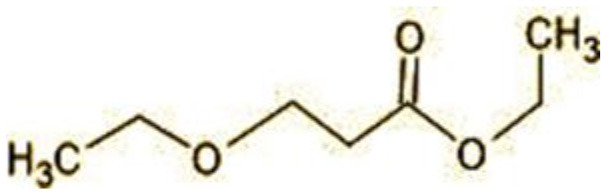		
7	77	3-hydroxy-3-phenyl propionic acid (C_9_H_10_O_3_)			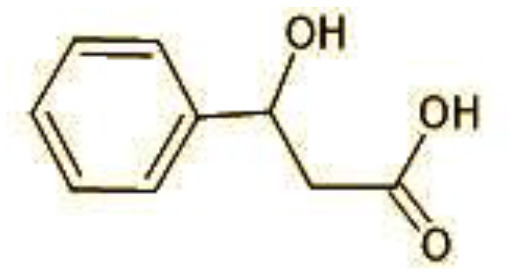	
8	98	Hexadecane (C_16_H_34_)				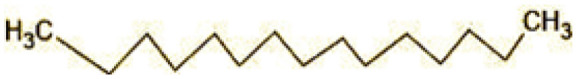
9	107	4-Hydroxybenzoic acid (C_7_H_6_O_3_)	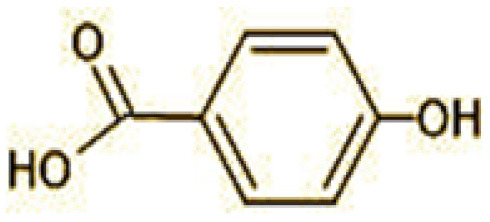			
10	119	p-isopropenylphenol (C_9_H_10_O)		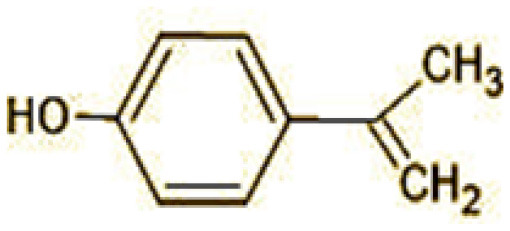		
11	137	4-ethyl-2-methoxyphenol (C_9_H_12_O_2_)				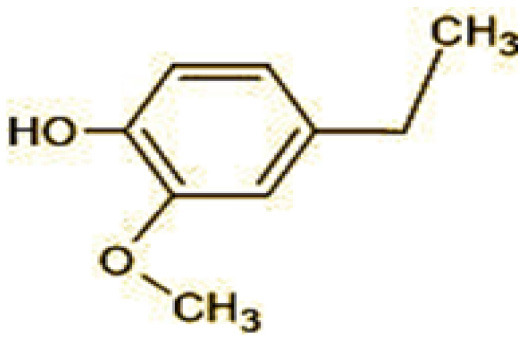
12	168	2-(2-hydroxypropyl)- benzene-1,4-diol (C_9_H_12_O_3_)			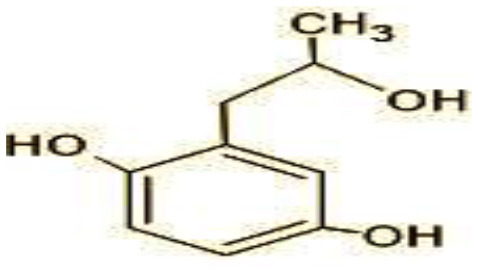	
**LC-MS/MS analysis of triclosan**						
1	287	Triclosan	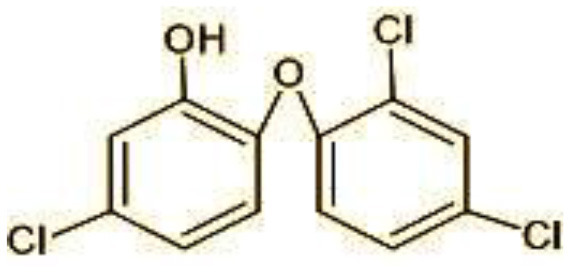			
2	144	4-Chlorocatechol (C_6_H_5_ClO_2)_		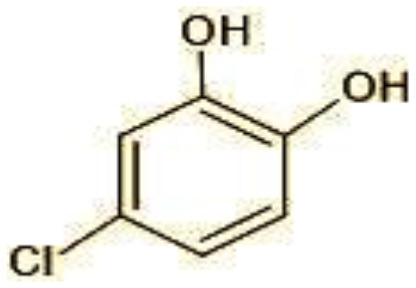		
3	165	2,4-Dichlorophenol (C_6_H_4_Cl_2_O)					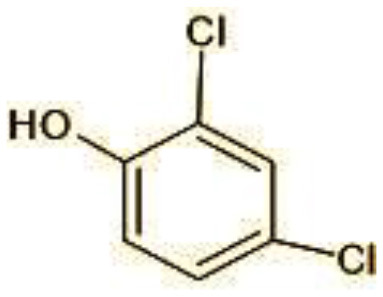
4	180	3,5-Dichlorocatechol (C_6_H_4_Cl_2_O_2_)			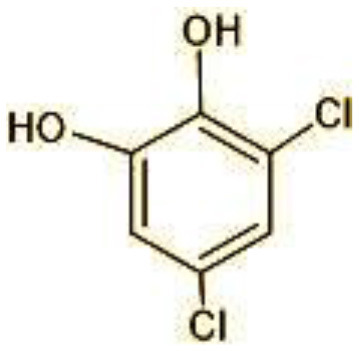		
5	192/194	2-Methoxy-3,5-dichlorophenol (C_7_H_6_Cl_12_O_2_)				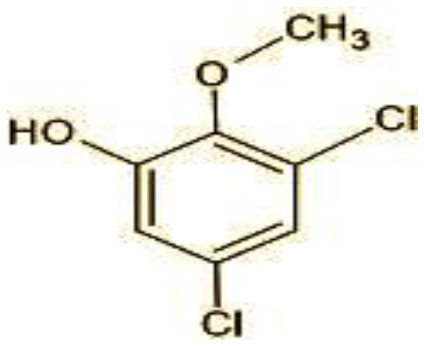	
6	215	2,4-dicholoro-3, 5,6-trihydroxyhexanol (C_6_H_10_C_l2_O_4_)	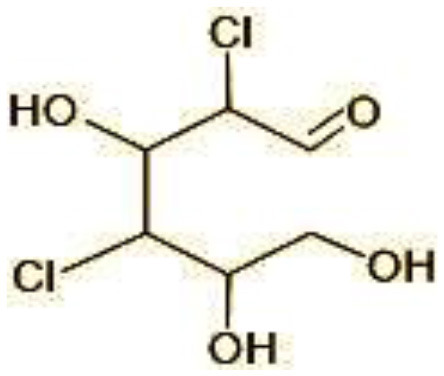				
7	249	5-mercapto-2-(4-mercaptophenoxy)phenol (C_12_H_10_O_2_S_2_)						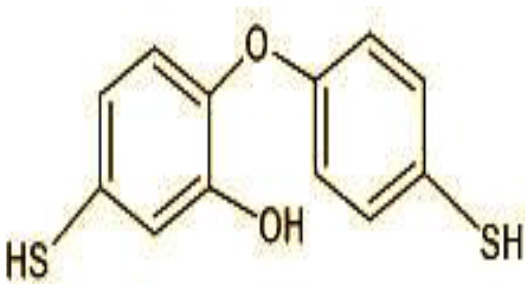
8	272	(E)-2-(2,4-dichlorophenoxy)-4-formylbut-2-enoic acid (C_11_H_8_Cl_2_O_4)_		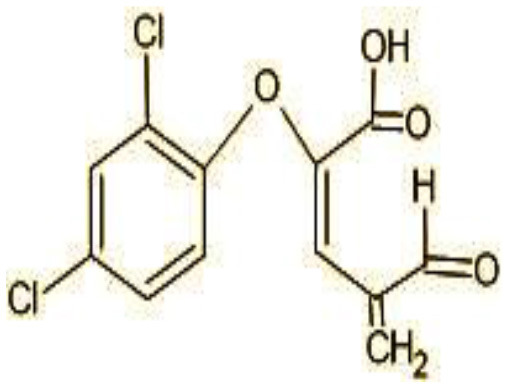				
9	273	2,4,-dichloro-2′3′-dihydroxydiphenyl ether (C_12_H_8_Cl_2_O_3_)					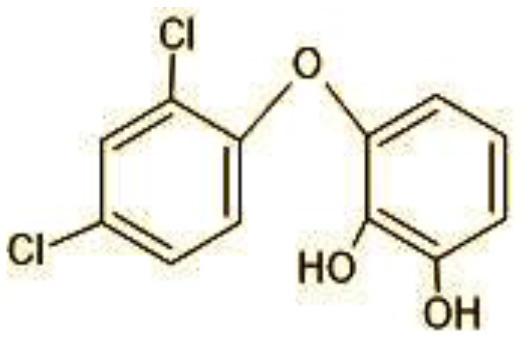	
10	337	Dihydroxy Triclosan (C_12_H_9_Cl_3_O_5_)							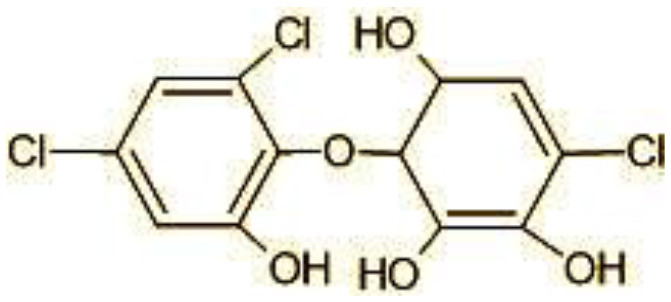
11	360/362	TMS Triclosan (C_18_H_12_Cl_2_OSi_4_)			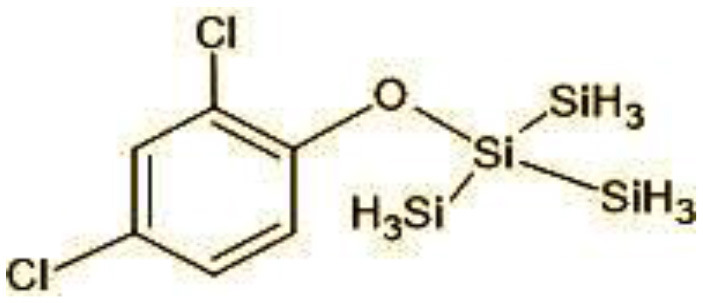				

The identification of metabolites formed after degradation of 50 ppm TCS on 20^th^ day (Supplementary Figures S8, S9) showed laccase catalyzed breaking of ether bond between two aromatic rings of TCS and introduction of hydroxy radicals. Triclosan possesses positions for an initial dihydroxylation in both the monochlorinated and dichlorinated aromatics rings leading to the formation of 2, 4-dichlorophenol (m/z = 165) in monochlorinated ring and 4-chlorocatechol (m/z = 144) in dichlorinated ring as was also evident by Kim et al. (2011). 2-4 DCP was observed to be major intermediate, which was then transformed into 3,5-dichlorocatechol (m/z = 181). Ortho-methylation of this phenol carrying electron attracting group resulted in the formation of 2-methoxy-3, 5-dichlorophenol (m/z = 192) which is a well-known mechanism to detoxify such potential hazardous compounds (Kim et al., 2011). Another metabolite with m/z = 215 was observed which would have been derived from 2,4-dichlorophenol (m/z =165) by the ring fission (Kim et al., 2011). The appearance of metabolite with m/z of 337 (dihydroxy triclosan) suggested its formation by hydroxylation of both aromatic rings as also reported by Zhao et al. (2012) and Wang et al. (2018). This was followed by a cleavage (ring opening) reaction resulting in the formation of metabolites with m/z of 195 and 225, respectively, that might be due to the hydrolases secreted by *H. lixii*. Metabolite with m/z = 273 (2,4,-dichloro-2′3′-dihydroxydiphenyl ether) was formed due to reductive dechlorination (hydrogenolysis) (Table 2, Figure 7B) (Wang et al., 2018). Thus, the transformation of TCS into less chlorinated metabolites justifies the detoxification mechanism resulting in decrease in toxicity levels.

### 3.9 Bioaccumulation

**Figure 8:**
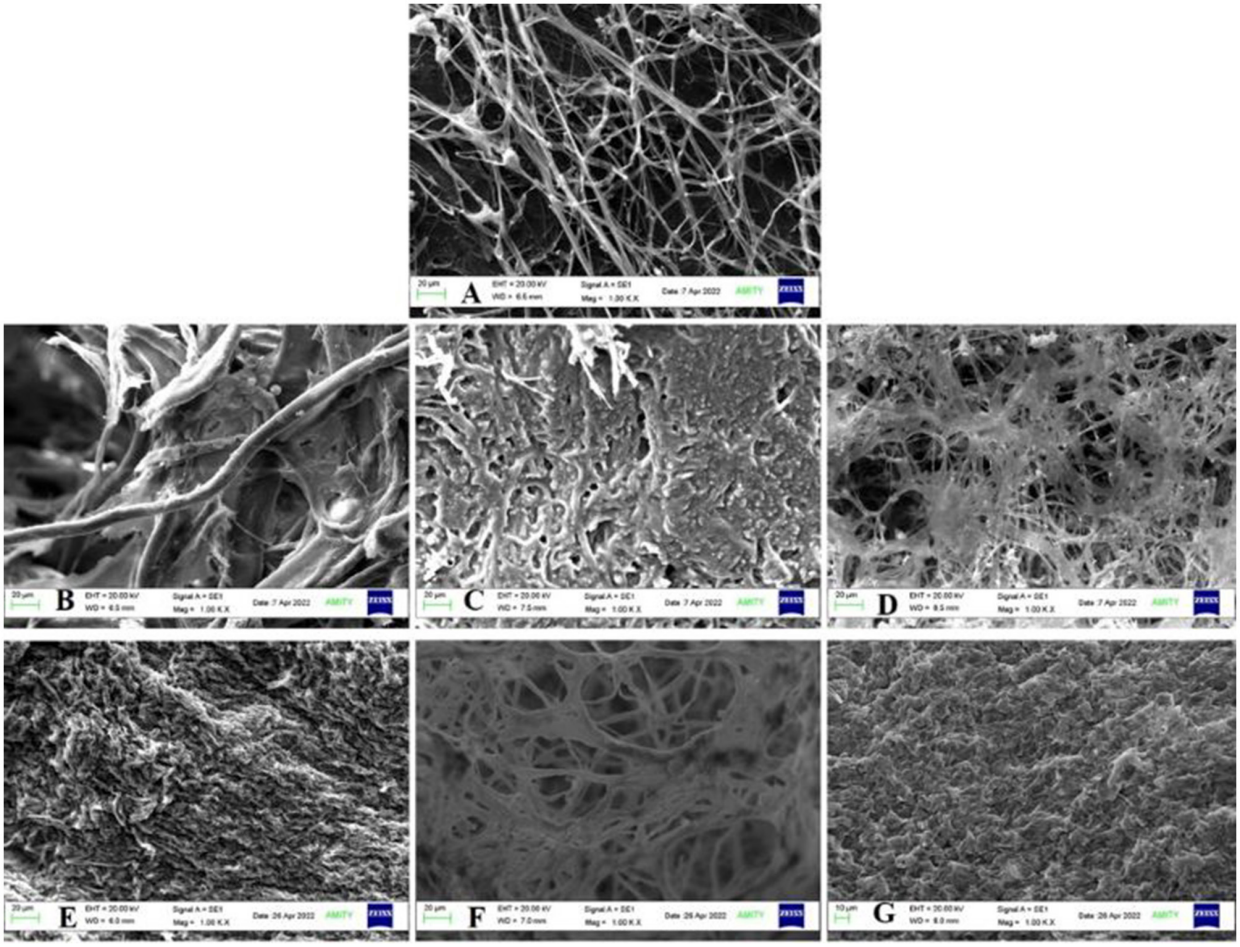
SEM analysis of bioaccumulation **(A)** Control; BPA—**(B)** 10 ppm, **(C)** 50 ppm, and **(D)** 100 ppm; TCS—**(E)** 10 ppm, **(F)** 50 ppm, and **(G)** 100 ppm.

Under static conditions, the accumulation of BPA in growing fungal mat was observed to increase with EDC concentration with 193.9 μg/g for 10 ppm, 272.22 μg/g for 50 ppm, and 777.07 μg/g for 100 ppm. Similarly, for triclosan, it was found to be 171.99 μg/g in 10 ppm, 189.16 μg/g in 50 ppm, and 516.88 μg/g in 100 ppm, respectively (Supplementary Figure S10). SEM of the fungal mycelium surface revealed irregular rough, granular surface due to swollen short branches at all concentrations as compared to the control (mycelia without EDC treatment) with long and rope-like highly branched smooth walled fungal hyphae (Figure 8).

**Figure 9:**
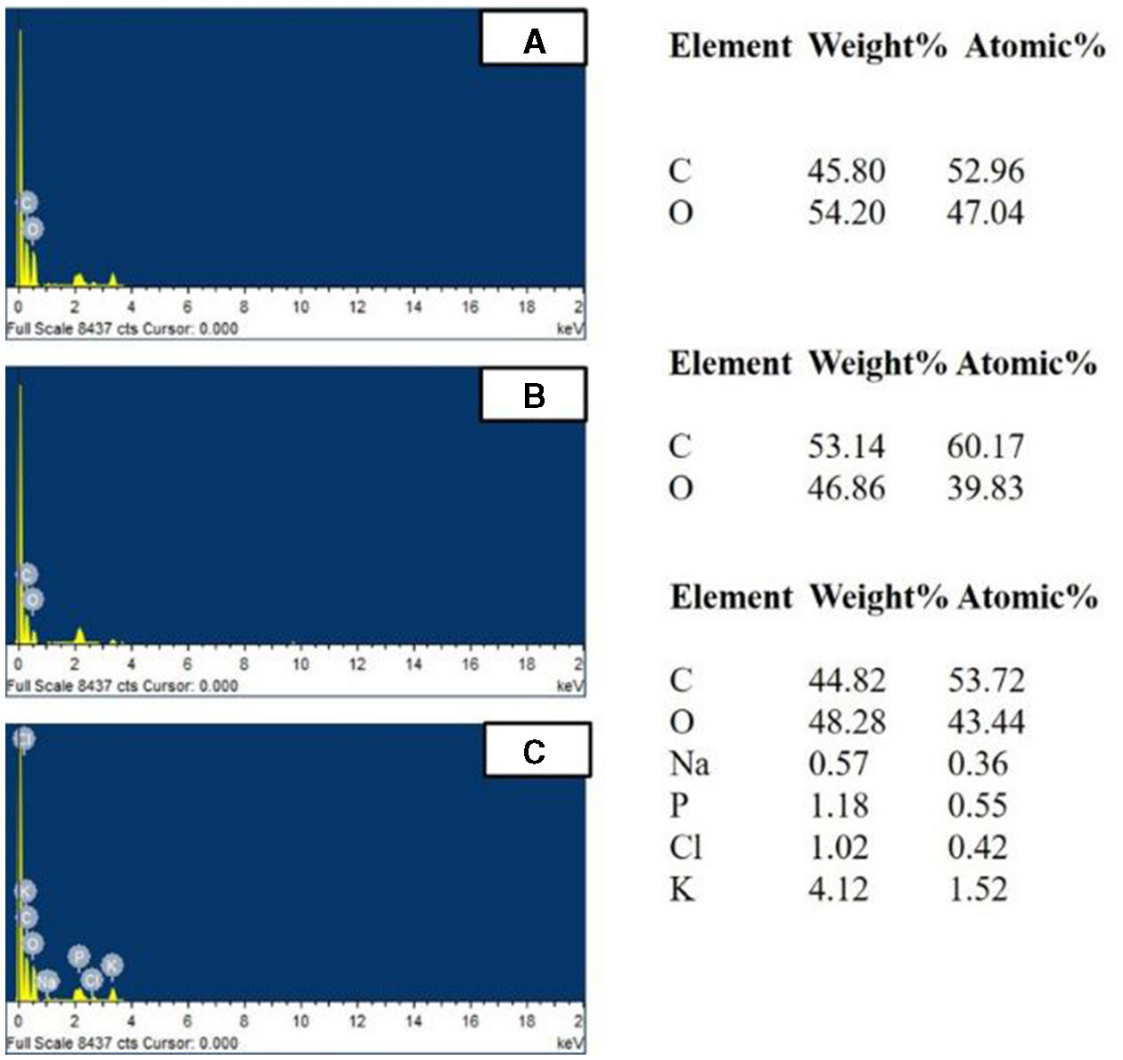
EDX analysis of *Hypocrea lixii* accumulated with/without **(A)** Control (without EDCs), **(B)** 100 ppm BPA, and **(C)** 100 ppm TCS.

The bioaccumulation was further confirmed by EDX which represented BPA accumulation by the increase in the percentage of carbon to 53.14% in the presence of 100 ppm BPA against the control (without BPA) with 45.80% (fungal biomass) (Figure 9) (Garikoé et al., 2022). Similarly, the increase in percentage of chlorine from 0% to 1.02%, a main component of triclosan, confirmed triclosan accumulation in EDX spectra (Triwiswara et al., 2020).

## 4 Conclusion

From our study, it can be concluded that under shake flask conditions, the sets wherein EDCs were added after 5 days of substantial growth of WRF S5 *Hypocrea lixii* (set B) favored better degradation than set A where EDCs were added at time of inoculation. The expression of LMEs was also triggered in the presence of EDC. The WRF isolate showed lowering of surface tension during the degradation process with 44.16 mN/m in case of BPA and 38.49 mN/m in TCS, respectively, and efficiently degraded bisphenol A (78.62%) and triclosan (69.18%). The FTIR, GC-MS, and LC-ESI-MS/MS confirmed the presence of surfactin lipopeptide isoforms. The biosurfactant formed during degradation also boosted the laccase and Lip units as compared to controls with no EDCs. At the end of degradation, pH of the culture filtrate of each degraded EDC was lowered to 4.5 and 4.8, supporting the formation of slightly acidic intermediate metabolites. The WRF isolate showed an immense potential to produce plant growth promoting traits as Zn solubilization, ACC deaminase activity, indole acetic acid, and siderophore production. These traits of WRF along with other microbes (bioaugmentation approach) could be adopted for remediation of other recalcitrant organic pollutants with the soil restoration of polluted sites.

## Data Availability

The datasets presented in this study can be found in online repositories. The names of the repository/repositories and accession number(s) can be found in the article/Supplementary material.
