# Supplementary material for: Solubilization and enhanced degradation of benzene phenolic derivatives—Bisphenol A/Triclosan using a biosurfactant producing white rot fungus Hypocrea lixii S5 with plant growth promoting traits
Source: Front Microbiol. 2024 Sep 18;15:1433745. doi: 10.3389/fmicb.2024.1433745 (PMC11445159; doi:10.3389/fmicb.2024.1433745)
Supplement: Supplementary file 1 [file Table_1.DOCX]

**Supplementary Information**

**Solubilization and enhanced degradation of benzene phenolic derivatives – Bisphenol A/ Triclosan using a biosurfactant producing white rot fungus *Hypocrea lixii* S5 with plant growth promoting traits**

Mridula Chaturvedi ^a1^; Navpreet Kaur^a^; Pattanathu K S M Rahman ^b^ and Shashi Sharma ^a^*

**^a^**Amity Institute of Biotechnology, Amity University, Sector- 125, Noida, UP-201313, India, **^b^**Centre for Natural Products and Discovery, School of Pharmacy and Biomolecular Sciences, Liverpool John Moores University, Liverpool, United Kingdom

**
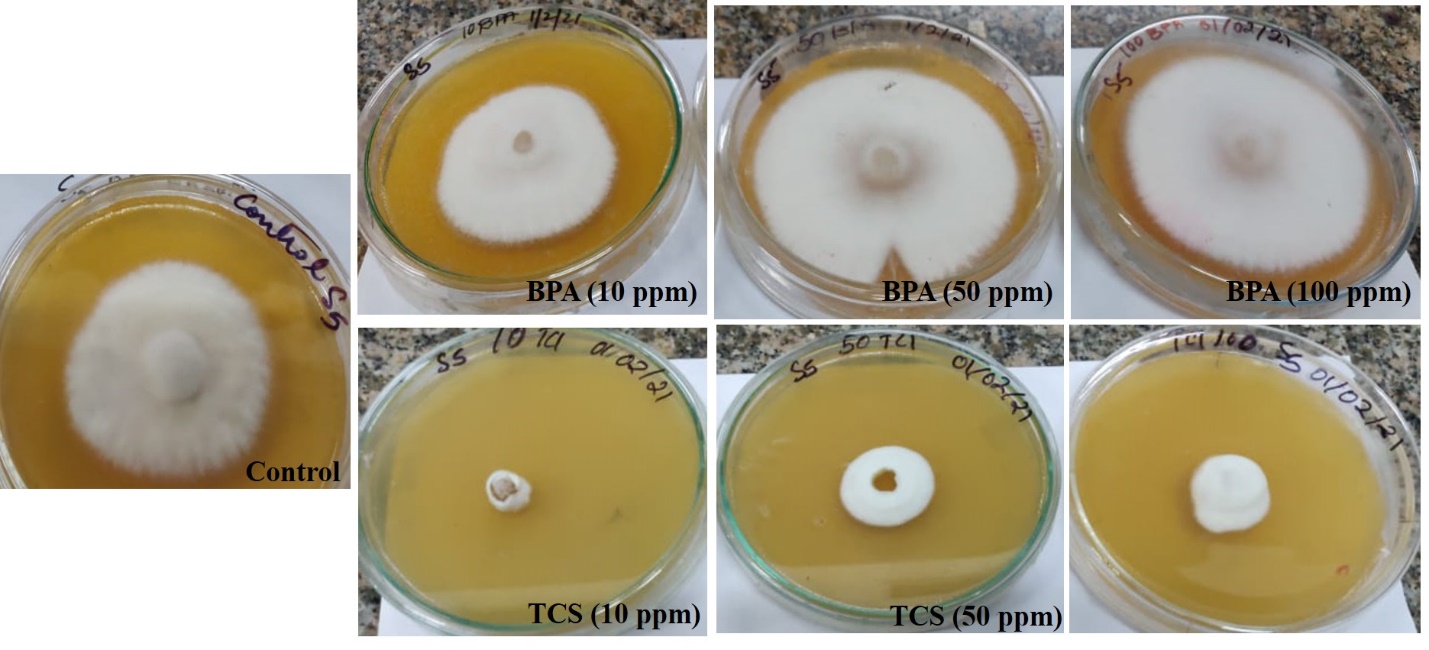
**

**Fig S1: Tolerance assay of isolate in different concentrations (10, 50 and 100 ppm) of EDCs**

**(Bisphenol A and Triclosan)**

**
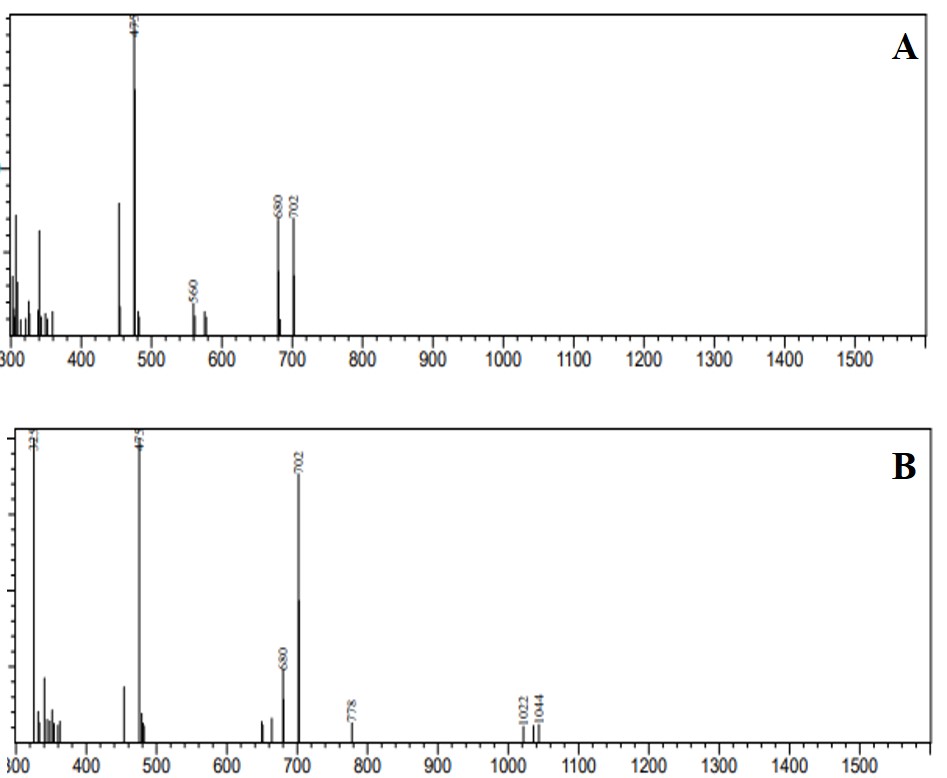
**

**Fig S2: LC-ESI-MS/MS analysis of biosurfactant produced from *Hypocrea lixii* in positive**

**ion mode**

**
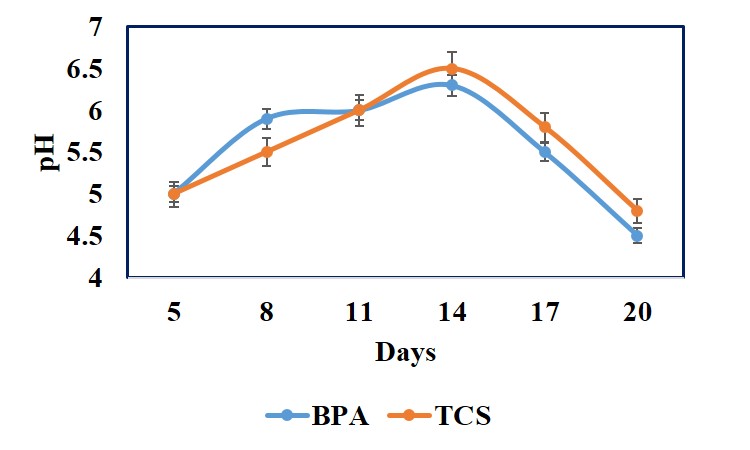
**

**Fig S3: Changes of pH during degradation of BPA and TCS**

**
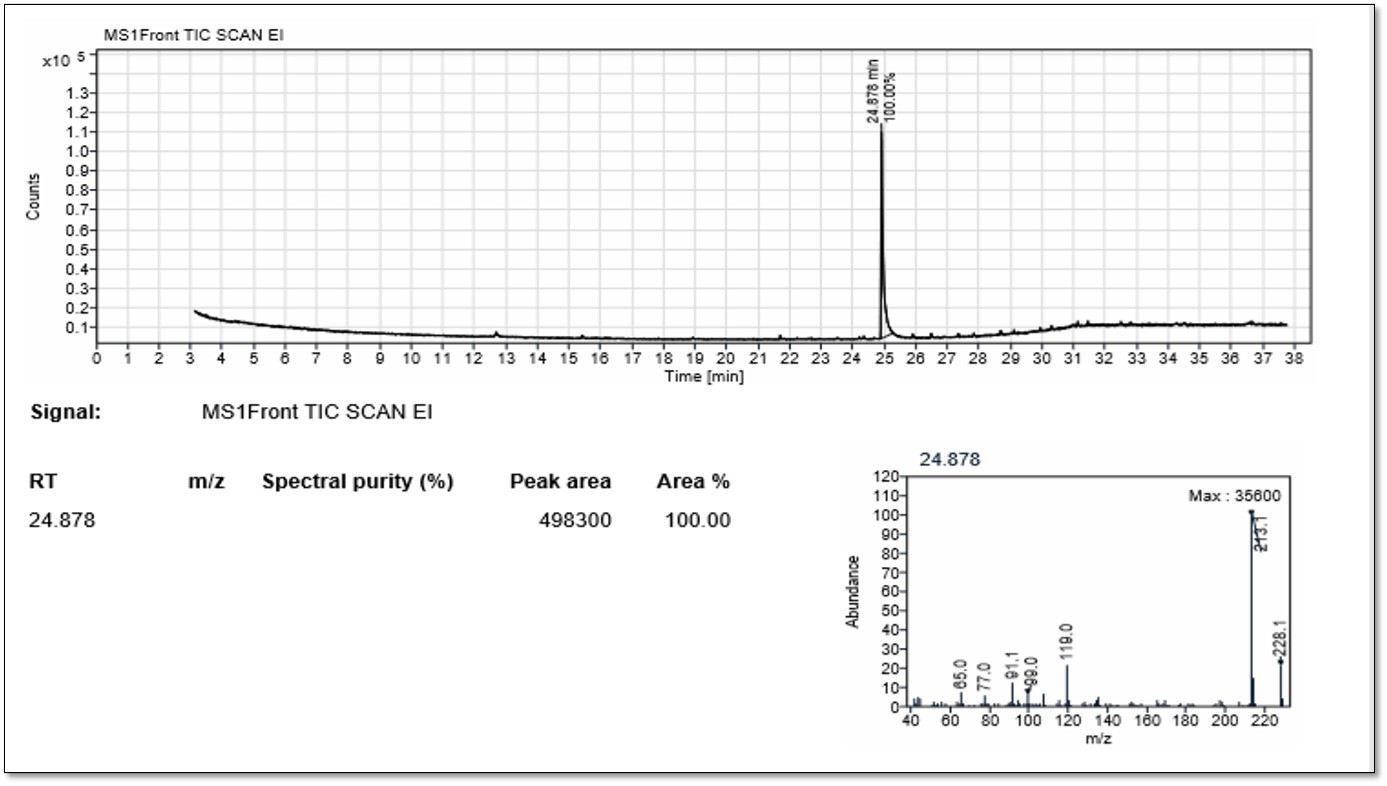
**

**Fig S4: Zero day degradation pattern of 50 ppm BPA (Set B) in synthetic medium**

**analyzed via GC- MS**


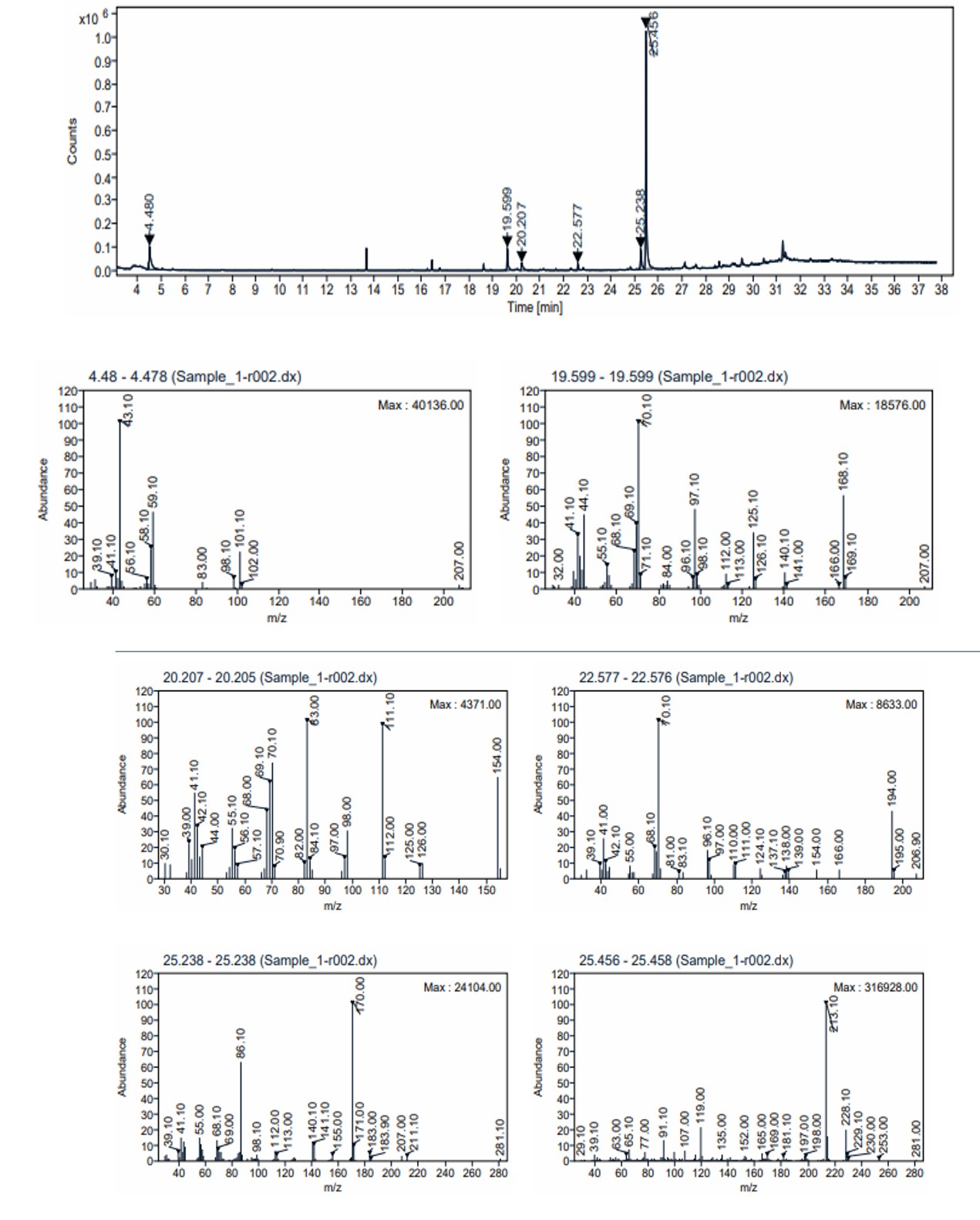
 **Fig S5: Residual analysis of 50 ppm BPA (Set B) by GC- MS on 11^th^ day in synthetic**

**medium**

**
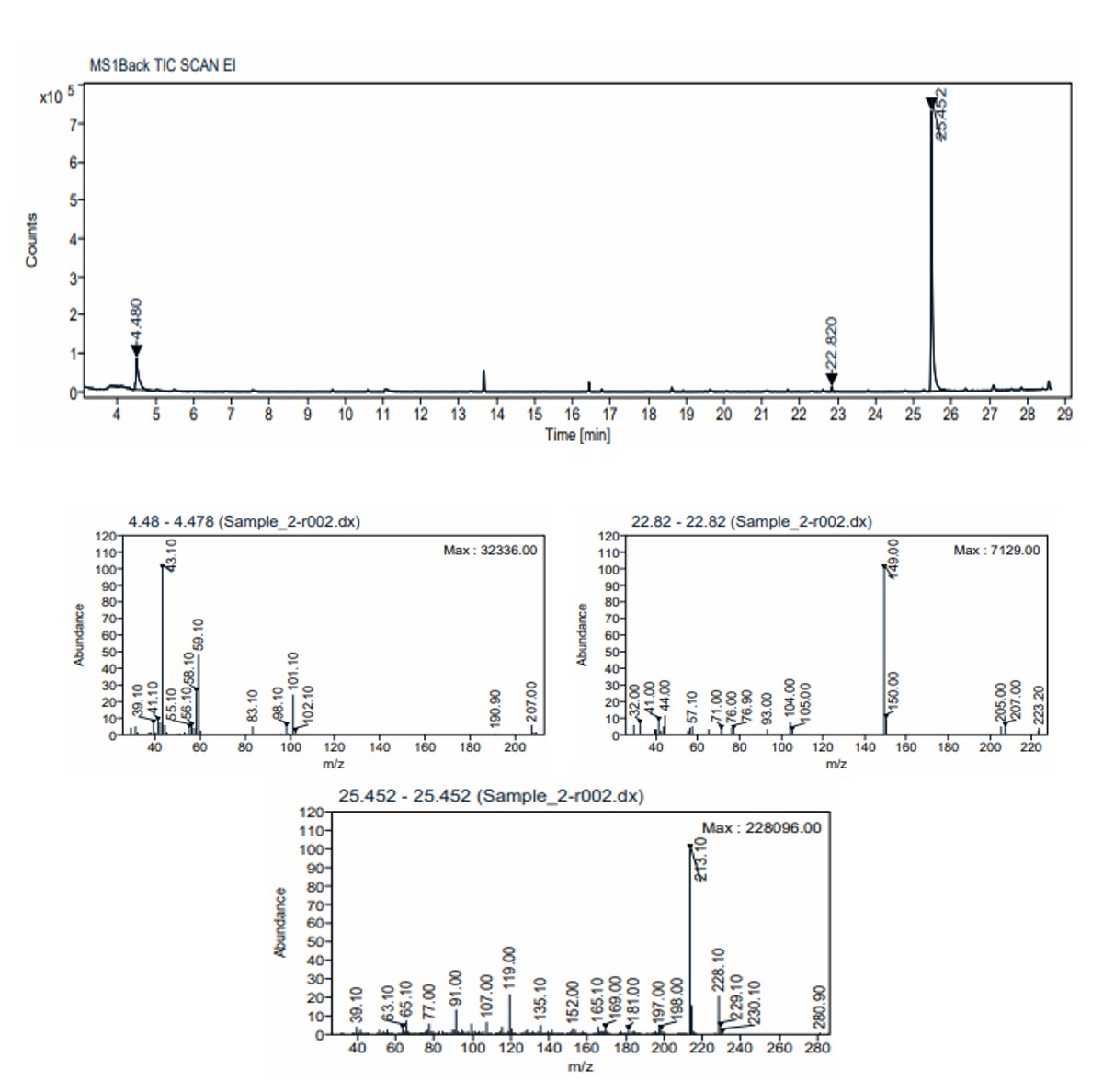
**

**Fig S6: Residual analysis of 50 ppm BPA (Set B) by GC- MS on 14^th^ day in synthetic**

**medium**

**
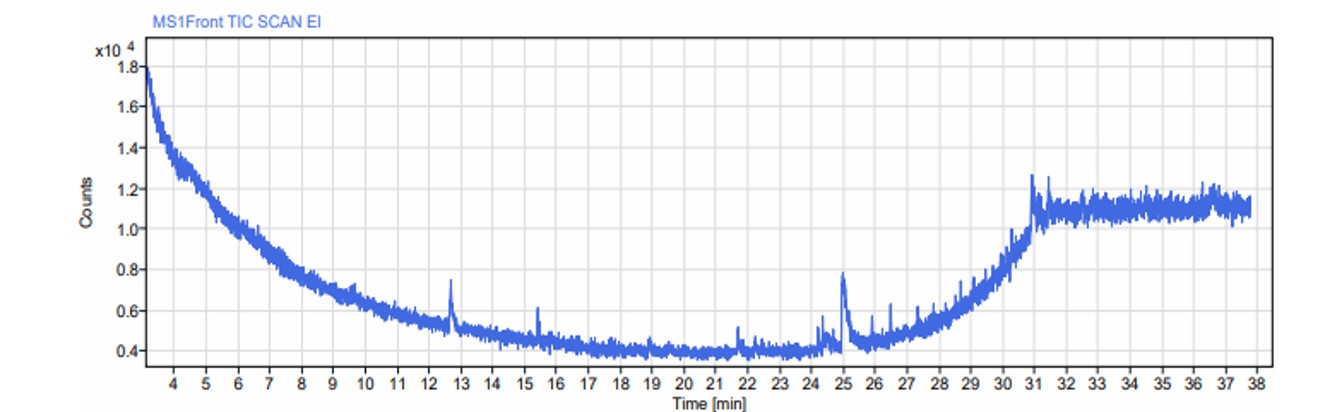
**

**Fig S7: Residual analysis of 50 ppm BPA (Set B) by GC- MS on 20^th^ day in synthetic**

**medium showed complete mineralization of BPA with no metabolites**

**
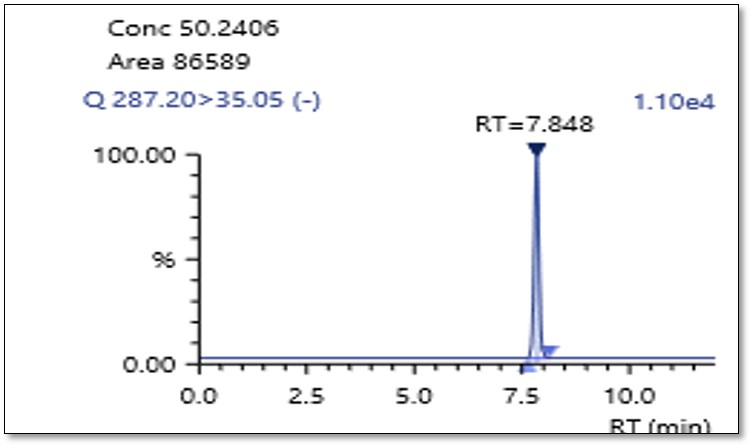
**

**Fig S8: Zero day degradation pattern of 50 ppm Triclosan (Set B) in synthetic medium via**

**LC- MS/MS**

**
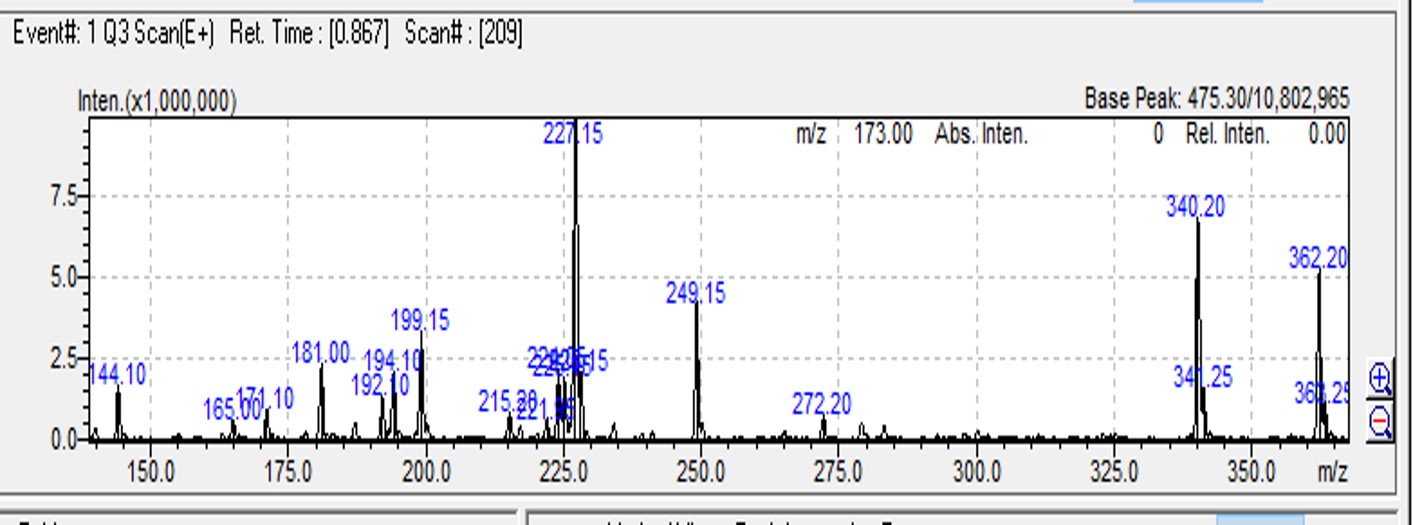
**

**Fig S9: Residual analysis of 50 ppm Triclosan (Set B) by LC- MS/MS on 20^th^ day in**

**synthetic medium**

**
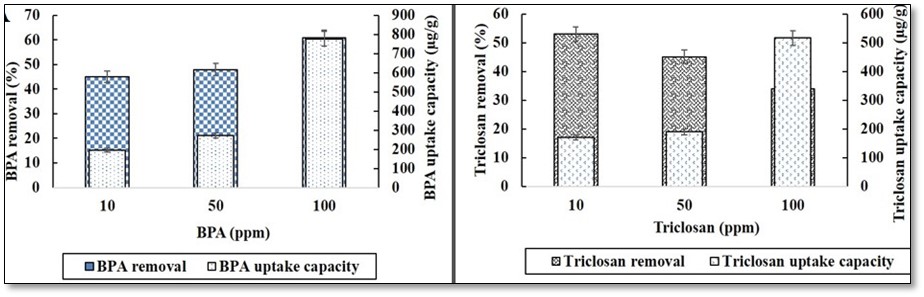
**

**Fig S10: Removal (%) and mycelial uptake capacity (µg/g) of BPA and Triclosan at 10, 50**

**and 100 ppm during bioaccumulation mode under static condition**
